# Overexpression of BQ323636.1 Modulated AR/IL-8/CXCR1 Axis to Confer Tamoxifen Resistance in ER-Positive Breast Cancer

**DOI:** 10.3390/life12010093

**Published:** 2022-01-10

**Authors:** Ho Tsoi, Ling Shi, Man-Hong Leung, Ellen P. S. Man, Zi-Qing So, Wing-Lok Chan, Ui-Soon Khoo

**Affiliations:** 1 Department of Pathology, Li Ka Shing Faculty of Medicine, The University of Hong Kong, Hong Kong, China; tsoiho@hku.hk (H.T.); u3005146@connect.hku.hk (L.S.); george09@connect.hku.hk (M.-H.L.); ellenman@hku.hk (E.P.S.M.); u3569644@connect.hku.hk (Z.-Q.S.); 2 Department of Clinical Oncology, Li Ka Shing Faculty of Medicine, The University of Hong Kong, Hong Kong, China; winglok@hku.hk

**Keywords:** breast cancer, BQ323636.1, tamoxifen resistance, androgen receptor, interleukin-8, CXCR1

## Abstract

NCOR2 is a co-repressor for estrogen receptor (ER) and androgen receptor (AR). Our group previously identified a novel splice variant of NCOR2, BQ323636.1 (BQ), that mediates tamoxifen resistance via interference of NCOR2 repression on ER. Luciferase reporter assay showed BQ overexpression could enhance the transcriptional activity of androgen response element (ARE). We proposed that BQ employs both AR and ER to confer tamoxifen resistance. Through in silico analysis, we identified interleukin-8 (IL-8) as the sole ERE and ARE containing gene responsiveness to ER and AR activation. We confirmed that BQ overexpression enhanced the expression of IL-8 in ER+ve breast cancer cells, and AR inhibition reduced IL-8 expression in the BQ overexpressing cell lines, suggesting that AR was involved in the modulation of IL-8 expression by BQ. Moreover, we demonstrated that IL-8 could activate both AKT and ERK1/2 via CXCR1 to confer tamoxifen resistance. Targeting CXCR1/2 by a small inhibitor repertaxin reversed tamoxifen resistance of BQ overexpressing breast cancer cells in vitro and in vivo. In conclusion, BQ overexpression in ER+ve breast cancer can enhance IL-8 mediated signaling to modulate tamoxifen resistance. Targeting IL-8 signaling is a promising approach to overcome tamoxifen resistance in ER+ve breast cancer.

## 1. Introduction

Breast cancer has long been the most prevalent cancer among women. In 2020, around 2.3 million women were diagnosed with breast cancer, accounting for 24.5% of all female cancer cases [1]. In 2020, breast cancer resulted in 685,000 female deaths, contributing to 15.5% of cancer deaths in women (World Health Organization, 2021). The incidence of breast cancer varies among different regions, ranging from 26.2 per 100,000 women in Central South Asia to 95.5 per 100,000 women in Australia/New Zealand [1]. The incidence of breast cancer is increasing on average 0.3% each year over 2009–2018. (National Cancer Institute, US, 2021). Based on gene expression profiles, breast cancer can be classified into five major subtypes, (1) luminal A, (2) luminal B, (3) HER2-enriched, (4) basal-like, and (5) normal-like [2]. The treatment of breast cancer is guided by the expression of estrogen receptor (ER), progesterone receptor (PR) and human epidermal growth factor receptor 2 (HER2). Usually, tamoxifen is used for ER-positive (ER+ve) breast cancer, Herceptin is used for HER2-overexpressed (HER2+ve) breast cancer, and chemotherapy is used for triple-negative breast cancer (TNBC).

About 75% of breast cancer patients belong to ER+ve [3]. ER is the primary tumor driver in this category. Estrogen promotes breast carcinogenesis by binding to ER to stimulate genomic and non-genomic activities essential for cancer-cell proliferation and growth [4]. The genomic pathway refers to ER activation within the cytoplasm to induce gene transcription via nuclear translocation. Upon binding to its ligand estrogen (e.g., estradiol), ER is dissociated from heat shock protein 70 (HSP70) and HSP90 [5]; activation of ER is the result. Activated ER forms homo-dimers, translocating to the nucleus, and directly binds to target genes with estrogen response elements (ERE). ER binding to its target genes recruits various co-activator proteins and components of RNA polymerase II transcription initiation complex, leading to enhanced ER-mediated gene transcription [4]. The non-genomic pathway refers to the activation of ER on the cell membrane. In this pathway, the binding of estrogen to membranous ER leads to activation of multiple downstream signaling pathways, including MAPK/ERK and PI3K/AKT pathways [4]

Targeting ER is the most effective approach for treating ER+ve breast cancer. Currently, there are mainly three categories of targeted therapies available for suppressing estrogen-mediated signaling: selective estrogen receptor mediators (SERMs), selective estrogen receptor degraders (SERDs) and aromatase inhibitors (AIs). SERMs such as tamoxifen serve as antagonists that compete with estrogen binding to ER [6] and therefore inhibit ER-mediated transcriptional activity. Tamoxifen is the most frequently used first-line adjuvant treatment, given its effectiveness and convenient administration. It has been shown that tamoxifen taken orally by ER+ve breast cancer patients significantly reduced the annual death rate by 31% [7].

Although tamoxifen can effectively suppress tumor growth, one-third of the ER+ve patients with 5-year tamoxifen treatment will eventually suffer from tumor recurrence [8]. Tamoxifen resistance is thus an obstacle for clinical treatment. The mechanism of tamoxifen resistance can be classified into de novo resistance and acquired resistance [9]. De novo resistance is usually due to intrinsic mutation or lack of ER and metabolic failure of converting tamoxifen to its active form. Acquired resistance, which occurs during tamoxifen treatment, is a major limitation of tamoxifen efficacy. Multiple mechanisms have been proposed, such as loss or modification in ER expression and function, altered co-regulator expression, and activation of signaling cascades. Most patients who develop acquired resistance have dysfunctional ER but still maintained ER expression [10]. Loss of ER function can be caused by acquired ER gene mutation, modification in ER epigenetics and abnormal splicing [11]. Alterations in the activities of co-activators and co-repressors can modulate tamoxifen resistance by affecting ER transcriptional activities. Overexpression of co-activator protein SRC-3 has been associated with increased resistance to tamoxifen [12]. The absence of co-repressor NCOR1 and NCOR2 compromises tamoxifen’s anti-cancer effect and results in resistance [13]. Overexpression of BQ323636.1 (BQ), a splice variant of NCOR2 [14], can compromise the suppressive role of NCOR2 on ER and lead to the activation of ER in a ligand-independent manner [15]. Activation of AKT and ERK1/2-mediated pathways has been shown to induce tamoxifen resistance [16,17]. Therefore, the mechanism of tamoxifen resistance is complex.

The therapeutic effect of tamoxifen relies on its ability to inhibit ER-mediated transcriptional activities. Alterations in ER expression, its co-regulators and downstream signaling pathways may all contribute to tamoxifen resistance. Therefore, ER is central to understanding the mechanism of tamoxifen resistance. Androgen receptor (AR) has also been proposed to modulate tamoxifen resistance [18]. Overexpression of AR could assist ER action by replacing the co-repressors, recruiting co-activators, or even serving as co-activator [19]. BQ is shown to be commonly expressed in primary breast tumor samples from our previous study. BQ overexpression is found in around 50% of patients, and these eventually develop tamoxifen resistance [15]. NCOR2 is a negative regulator of ER [20] and AR [21]. BQ is a truncated form of NCOR2. When present in excess, BQ binds to NCOR2, resulting in the formation of a defective co-repressor complex for repression of transcription factor activation [15]. Overexpression of BQ should therefore enhance the transcriptional activities of ER and AR. We proposed that overexpression of BQ would significantly enhance the expression of genes with ERE and ARE. Through in silico analysis, we found that the interleukin-8 (IL-8) promoter contained both functional ARE and ERE. We showed that BQ overexpression could enhance the production of IL-8, which in turn activates the AKT and ERK1/2 signaling cascades via its receptor CXCR1. Inhibition of CXCR1 could compromise tamoxifen resistance in BQ overexpressing breast cancer. These results suggest that targeting IL-8 mediated signaling would be a possible approach to reduce tamoxifen resistance in ER+ve breast cancer.

## 2. Materials and Methods

### 2.1. Cell Cultures, Transfection and Stable Cell Lines Establishment

Human non-tumorigenic breast cell line MCF-10A and human breast cell lines MCF-7 and ZR-75 (tamoxifen-sensitive cell lines) were purchased from American Type Culture Collection (ATCC, Manassas, VA, USA). They were reauthenticated by short tandem repeat profiling [22]. LCC2 is a tamoxifen-resistant cell line derived from MCF-7, kindly provided by Dr. Robert Clarke (Georgetown University Medical School, Washington, DC, USA) and used in our previous study [22]). MCF-10A was cultured in DMEM/F12 (11330032; Thermo Fisher Scientific, Waltham, MA, USA) with 5% horse serum (16050122; Thermo Fisher Scientific, Waltham, MA, USA), 20 ng/mL of EGF (PHG0313; Thermo Fisher Scientific, Waltham, MA, USA), 0.5 mg/mL of Hydrocortisone (H-0888; Sigma-Aldrich, St. Louis, MO, USA), 100 ng/mL of Cholera Toxin (C-8052; Sigma-Aldrich, St. Louis, MO, USA), 10 μg/mL of insulin (I-1882; Sigma-Aldrich, St. Louis, MO, USA) and 1% penicillin/streptomycin (P/S; 10378016; Thermo Fisher Scientific, Waltham, MA, USA). MCF-7 and LCC2 cells were cultured and maintained in DMEM (12100046; Thermo Fisher Scientific, Waltham, MA, USA) supplemented with 10% fetal bovine serum (FBS; 26140079; Thermo Fisher Scientific, Waltham, MA, USA) and 1% P/S (10378016; Thermo Fisher Scientific, Waltham, MA, USA). ZR-75 cells were grown in Improved Minimum Essential Medium (IMEM, A104890; Thermo Fisher Scientific, Waltham, MA, USA) with 10% FBS and 1% P/S. All the cell lines were cultured in a tissue culture incubator with 5% CO_2_ at 37 °C. Cell lines used were confirmed mycoplasma-free. Mycoplasma screening was conducted by the Faculty Core Facility (Li Ka Shing Faculty of Medicine, The University of Hong Kong, Hong Kong, China) to ensure the cell culture was free from mycoplasma. Lipofectamine 2000 (11668019; Thermo Fisher Scientific, Waltham, MA, USA) was employed for the transfection of plasmids according to the manufacturer’s instruction. Oligofectamine (12252011; Thermo Fisher Scientific, Waltham, MA, USA) was used for the transfection of siRNA according to the manufacturer’s instructions. After 72 h post-transfection, 0.5 µg/mL of puromycin (A1113802; Thermo Fisher Scientific, Waltham, MA, USA) was employed for the selection of transfected cells. Fresh DMEM or IMEM with 10% FBS, 1% P/S and 0.5 µg/mL of puromycin was replaced every 72 h. The selection was performed for 6 weeks. The cell lines were maintained in DMEM or IMEM with 10% FBS, 1% P/S and 0.5 µg/mL of puromycin.

### 2.2. Plasmids and siRNA

We used pcDNA3.1-His-BQ323636.1. CXCR1 human shRNA plasmid kit (TL312158; Origene, Rockville, MD, USA) was employed. CXCR1 shRNA (sc-40026; Santa Cruz Biotechnology, Dallas, TX, USA), GFP control (sc-108084; Santa Cruz Biotechnology, Dallas, TX, USA) and control shRNA (sc-108080) were purchased. We purchased siRNA against IL-8 (L-004756) and non-targeting siRNA (D-001810) from Horizon Discovery (Cambridge, UK). We purchased siBQ.1 (5′-CUU CUC CAG GUU CUC UGC AUG-3′) and siBQ.2 (5′-CUC CAG GUU CUC UGC AUG CGC-3′) (Sigma-Aldrich, St. Louis, MO, USA) and described them in the published study [22].

### 2.3. Chemicals

We dissolved 4-hydroxytamoxifen (TAM; H7904; Sigma-Aldrich, St. Louis, MO, USA) in ethanol or DMSO at 5 mM as stock concentration. CXCR1 inhibitor repertaxin (S8640; Selleckchem, Houston, TX, USA) was dissolved in ethanol at 50 mM as stock concentration. Estradiol (E2; E8875; Sigma-Aldrich, St. Louis, MO, USA) was dissolved in ethanol at 1 μM. Dihydrotestosterone (DHT; D-073; Sigma-Aldrich, St. Louis, MO, USA) was dissolved in methanol at 1 mg/mL. Recombinant interleukin-8 (IL-8; 208-IL; R&D Systems, Inc., Minneapolis, MN, USA) protein was purchased and dissolved in double-distilled water. Bovine Serum Albumin (BSA; A7030; Sigma-Aldrich, St. Louis, MO, USA) was purchased and dissolved in double-distilled water. Bicalutamide (S1190; Selleckchem, Houston, TX, USA) was dissolved in DMSO at 100 mM as stock concentration.

### 2.4. Cell Viability Assay

MTT assay (M6494; Thermo Fisher Scientific, Waltham, MA, USA) was performed, and 5000 cells were seeded in 96-well plate. A clonogenic assay was performed, and 2000 cells were seeded in 12-well plate. We used 0.01% of crystal violet (C0775; Sigma-Aldrich, St. Louis, MO, USA) to stain the cell colonies. Cells were seeded on 24 wells. The colonies were stained with 0.01% crystal violet and counted under a microscope. A colony with more than 50 cells was regarded as a colony. All experiments were performed in triplicate. Microplate reader Infinite F200 (Tecan, Seestrasse, Switzerland) was used to record the absorbance.

### 2.5. RNA Extraction, Reverse Transcription and qPCR

RNA was isolated using TRIzol (15596026; Thermo Fisher Scientific, Waltham, MA, USA) according to the manufacturer’s instructions. Briefly, 1 × 10^6^ cells were homogenized and lysed in 400 µL TRIzol reagent and mixed with 200 µL chloroform. The aqueous phase that contains the RNA was obtained by centrifugation. RNA was subsequently precipitated with isopropanol and washed with 75% ethanol. RNA was solubilized in DEPC-treated water and the concentration was measured by NanoDrop 1000 (Thermo Fisher Scientific, Waltham, MA, USA). A cDNA synthesis was performed using PrimeScript^TM^ RT Master Mix (RR036B; Takara Biomedical Technology Co., Ltd., China). Briefly, 0.5 µg of RNA was mixed with 5X PrimeScript RT Master Mix and RNase-free water up to 10 µL total volume and incubated at 37 °C for 15 min, and the reaction was heat inactivated at 85 °C for 5 s. The synthesized cDNA was diluted with 40 µL RNase-free water. Samples of cDNA were analyzed by qPCR using the StepOne Real-Time PCR system (Thermo Fisher Scientific, Waltham, MA, USA). Reactions were performed in 10 µL volumes with diluted cDNA, PowerUp™ SYBR™ Green Master Mix (A25742; Thermo Fisher Scientific, Waltham, MA, USA) and qPCR primers. The qPCR primers were shown in the Supplementary Table (Table S1). The ΔΔCT method was employed to determine the relative expression of a gene.

### 2.6. Western Blot

Cells were lysed in cell lysis buffer (9803; Cell Signaling Technology, Danvers, MA, USA) supplemented with cOmplete Mini, EDTA-free protease inhibitor cocktail tablets (4693159001; Sigma-Aldrich, St. Louis, MO, USA), phenylmethanesulfonyl fluoride (PMSF; P7626; Sigma-Aldrich, St. Louis, MO, USA) and PhosSTOP EASYPack tablets (4906837001; Sigma-Aldrich, St. Louis, MO, USA). DC protein assay kit (5000112; Bio-Rad, Hercules, CA, USA) was employed to determine protein concentrations. A 20 μg of protein sample was separated using SDS-PAGE. Proteins were transferred to PVDF (1620177; Bio-Rad, Hercules, CA, USA). The following antibodies were used: anti-p-ERK1/2 (Thr202/Tyr204) (1:1000; 9102; Cell Signaling Technology, Danvers, MA, USA), anti-p-AKT (Ser473) (1:1000; 9106; Cell Signaling Technology, Danvers, MA, USA), anti-AKT (1:1000; 9272; Cell Signaling Technology, Danvers, MA, USA), anti-p-AKT (1:1000; 4058; Cell Signaling Technology, Danvers, MA, USA), anti-HIS tag (1:5000; 018-23224; FUJIFILM Wako Pure Chemical Corporation, Osaka, Japan), anti-CXCR1 (1:1000; PA5-95749; Thermo Fisher Scientific, Waltham, MA, USA), anti-GAPDH (1:5000; sc-365062; Santa Cruz Biotechnology, Dallas, TX, USA), anti-actin (1:5000; sc-47778; Santa Cruz Biotechnology, Dallas, TX, USA). The following secondary antibodies were used: anti-mouse HRP (1:5000; P0447; Aligent Dako, Santa Clara, CA, USA), anti-rabbit HRP (1:5000; P0260; Aligent Dako, Santa Clara, CA, USA). Densitometry readings/intensity ratio of each band (Figure S5) calculated for analysis by ImageJ (National Institutes of Health, Bethesda, MD, USA) and the original blots were shown in Figure S6.

### 2.7. IL-8 Measurement and AKT Activity Assay

The IL-8 Human ELISA Kit (KHC0081; Thermo Fisher Scientific, Waltham, MA, USA) was employed to determine the amount of interleukin-8 in the culture medium; 50 µL of the culture medium was used. Absorbance at 450 nm was recorded by microplate reader Infinite F200 (Tecan, Seestrasse, Switzerland). AKT Kinase Activity Assay Kit (ab139436; Abcam, Cambridge, UK) was used for determining AKT kinase activity. The signal was developed according to the manufacturer’s instructions. Absorbance at 450 nm was recorded by microplate reader Infinite F200 (Tecan, Seestrasse, Switzerland).

### 2.8. Luciferase Reporter Assay

AR transcriptional activity was determined by ARE-luciferase (ARR3tk-eGFP/SV40-mCherry; 132360; Addgene, Watertown, MA, USA) [23]. ER transcriptional activity was determined by ERE-luciferase (3X ERE TATA luc; 11354; Addgene, Watertown, MA, USA) [24]. The pCMV-Green Renilla Luc vector (pCMV-Ren; 16153; Thermo Fisher Scientific, Waltham, MA, USA) was used for normalization. The ratio of ARR3tk-eGFP/SV40-mCherry to pCMV-Ren and 3X ERE TATA luc to pCMV-Re*n* was 100:1. Dual-Luciferase^®^ Reporter Assay System (E1910; Promega, Madison, WI, USA) was used, and the signal was captured and recorded by microplate reader Infinite F200 (Tecan, Seestrasse, Switzerland).

### 2.9. Xenograft

Female nude mice at the age of 5 to 6 weeks were used for this study. On the day of inoculation, the cell mixture containing 1 × 10^6^ ZR-75-BQ cells was implanted into the mice’s abdominal mammary fat pad. The cell mixture was prepared by mixing 50 µL of the cell suspension containing 1 × 10^6^ cell with 50 µL of Matrigel (356234; BD Bioscience, Franklin Lakes, NJ, USA), and the 100 µL of the cell mixture was injected into the mammary fat pad. When the tumors were palpable, mice were randomized into 5 groups: (1) Saline (*n* = 4); (2) 4-OHT (N = 4); (3) 15 mg/Kg repertaxin (*n* = 4); (4) 4-OHT + 15 mg/Kg repertaxin (*n* = 4) and; (5) 4-OHT + 15 mg/Kg repertaxin (*n* = 4). 0.5 mg of tamoxifen dissolved in peanut oil (Sigma-Aldrich, St. Louis, MO, USA) and/or repertaxin (7.5 mg/kg and 15 mg/Kg) by subcutaneous injection twice per week. The tumor sizes were measured regularly using a caliper, and the tumor volume was calculated as the longest diameter x (shortest diameter)^2^/2. At the endpoint of the experiments, mice were euthanized, and tumors were harvested. All the procedures were reviewed and approved by the HKU Committee on the Use of Live Animals in Teaching and Research (CULATR Number: 5140-19).

### 2.10. Immunohistochemistry

Tissue microarray analysis (TMA) was approved by the Institutional Review Board of The University of Hong Kong/Hospital Authority Hong Kong West Cluster (HKU/HA HKW IRB No. UW 08-147). Histological sections were reviewed by the pathologist. For each case, donor blocks were chosen from the representative paraffin tumor blocks, and the selected region was marked for the construction of the TMA block. Clinical data were retrieved from the Department of Pathology, Queen Mary Hospital of Hong Kong. A total of 137 cases (Table S2) were used for scoring of BQ323636.1 and CXCR1 staining. Each case was constructed as triplicates, and the average score was used for the case. TMA sections were deparaffinized by xylene incubation and rehydrated by ethanol. Antigen retrieval was completed by using 0.01 M citrate buffer (pH 6). The slides were put into 3% H_2_O_2_ to quench endogenous peroxidase. The slides were rinsed with PBST twice, followed by incubation with primary monoclonal CXCR1 antibody (1:200; MA 1-206; Thermo Fisher Scientific, Waltham, MA, USA) and BQ323636.1 antibody (1:50; D-12, Veritech Ltd., Hong Kong, China) at 4 °C overnight. The slides were further washed by PBST and incubated with Envision + System- HRP Labelled Polymer Anti-Mouse (1:500; K4001; Aligent Dako, Santa Clara, CA, USA) for 30 min at room temperature. The slides were then washed by PBST, followed by incubation with chromogen DAB/substrate reagent for 1 min. After dehydration, the slides were mounted. TMA slides were visualized by the Aperio ScanScope system (Leica Biosystems, Wetzlar, Germany). Two individuals were assigned to finish the scoring. Cytoplasmic expression of CXCR1 was scored, whereas nuclear expression was scored for BQ. The intensity of cytoplasmic CXCR1 was scored as follows: 1 = weak, 2 = moderate, 3 = strong. For the percentage of staining, it was scored as follows: 1 ≤ 25%, 2 ≤ 50%, 3 ≤ 75%, 4 > 75%. The final score was calculated as follows: the score of intensity x the score of percentage. The H-score for nuclear expression was used for BQ323636.1, calculated as follows: 1x% of cells stained at “low” intensity + 2x% of cells stained at “moderate” intensity + 3x% of cells stained at “high” intensity. The median of the H-score was set as the threshold, which was 110 for nuclear BQ and 6.667 for cytoplasmic CXCR1.

### 2.11. In Silico Analysis

Transcriptional Regulatory Element Database (http://rulai.cshl.edu/cgi-bin/TRED/tred.cgi?process=home; assessed on 18 May 2019) was used to identify genes with androgen response element (ARE) and estrogen response element (ERE), which are androgen receptor targeted-gene and estrogen receptor-targeted gene, respectively. The default setting was used. Lists of genes targeted by AR and ER were retrieved. Genes with both ERE and ARE were identified by comparing the two lists of target genes.

### 2.12. Statistical Analysis

All numerical data were processed in Excel (Microsoft), Prism5 (GraphPad) or SPSS25 (IBM). Data were expressed as mean ± SD from at least three independent experiments. The Mann–Whitney U test or the Students’ *t*-test were performed to compare the variables of the two sample groups. One-way ANOVA and two-way ANOVA were employed to determine the statistical significance for multiple groups. The statistical significance between any two groups was determined by Bonferroni’s multiple comparison test. All tests were two-sided unless otherwise specified. Chi-square (χ^2^) test was used for hypothesis testing. Correlation with survival study of Tissue Microarray expression data was analyzed by Kaplan–Meier estimates followed by the log-rank test carried out by SPSS. Cox proportional hazards regression was used to estimate the association between clinical-pathological parameters, or BQ and CXCR1 scores with survival. Relative risk (RR) and 95% confidence interval (CI) were reported. The proportional-hazards assumption was tested using the Omnibus test, and no major model violation was observed. We considered *p* < 0.05 considered statistically significant; *, **, and *** represent *p* < 0.05, *p* < 0.01 and *p* < 0.001.

## 3. Results

### 3.1. Overexpression of BQ Could Activate AR Signalling and Thus Modulate the Response to Tamoxifen in Breast Cancer

**Figure 1:**
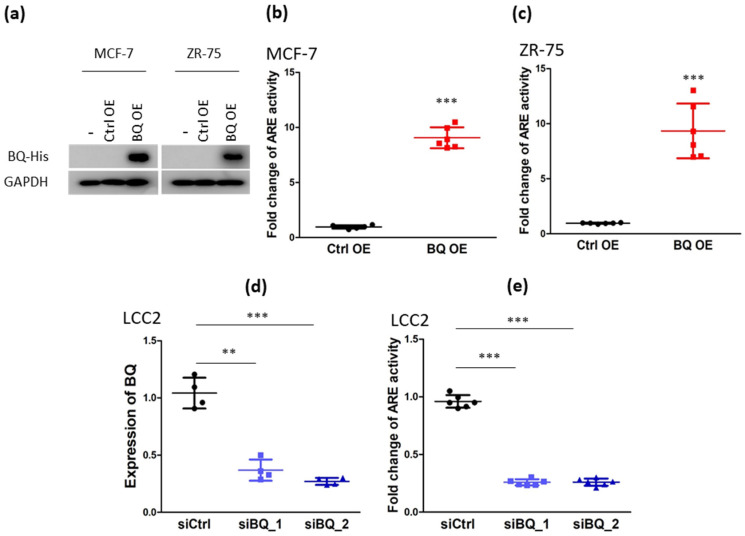
Overexpression of BQ323636.1 (BQ) can modulate the activity of androgen receptor (AR) in breast cancer cells. (**a**) Western blot was employed to confirm BQ ectopic expression in stable BQ overexpressing MCF-7 and ZR-75 cell lines. GAPDH was used as the loading control. Overexpression of BQ could enhance the transcriptional activity of AR in (**b**) MCF-7 and (**c**) ZR-75. Luciferase reporter assay with androgen response element (ARE) was employed. Results were shown as mean ± SD from 6 independent experiments. Student’s *t*-test was employed to determine statistical significance. *** represents *p* < 0.001. (**d**) Knockdown efficiency of siRNAs against BQ. LCC2 was transfected with 25 µM of non-targeting siRNA (siCtrl), siBQ.1 or siBQ.2. qPCR was performed 72 h post-transfection. Actin was used as the internal control. Results were shown as mean ± SD from 4 independent experiments. One-way ANOVA was employed. Bonferroni’s multiple comparison test was employed to determine the significance between 2 groups. ** and *** represent *p* < 0.01 and *p* < 0.001 respectively. (**e**) Knockdown of BQ could reduce AR activity in LCC2. Luciferase reporter assay with ARE was used. Results were shown as mean ± SD from 6 independent experiments. One-way ANOVA was employed. Bonferroni’s multiple comparison test was employed to determine the significance between 2 groups. *** represents *p* < 0.001.

High expression of BQ was found in the tamoxifen-resistant LCC2 cell line (Figure S1a; Figure S6e), which was consistent with our previous studies [15,23]. BQ interacted with NCOR2 and compromised the repressor function of NCOR2 [15]. Since NCOR2 is a repressor of estrogen receptor α (ER), the presence of excess BQ could suppress its repressive activity, leading to the activation of ligand-independent activation of ER signaling [15]. NCOR2 is also a repressor of androgen receptor (AR) [21]. Therefore, we speculated that overexpression of BQ (Figure 1a; Figure S6a) would induce the activation of AR signaling in breast cancer cells. Through the luciferase reporter assay, we confirmed that BQ overexpression could enhance the transcriptional activity of the androgen response element (ARE) in MCF-7 (Figure 1b) and ZR-75 cells (Figure 1c). Similarly, we found that ARE activity in LCC2 was significantly higher than that in MCF-7 (Figure S1b). Next, we determined the effect of BQ down-regulation (Figure 1d) on ARE activity in LCC2, a tamoxifen-resistant cell line with high expression of endogenous BQ. The results from the reporter assay indicated that knockdown of BQ could reduce ARE activity in LCC (Figure 1e). These results suggest that BQ can modulate the activity of the AR-driven pathway in breast cancer cells.

**Figure 2:**
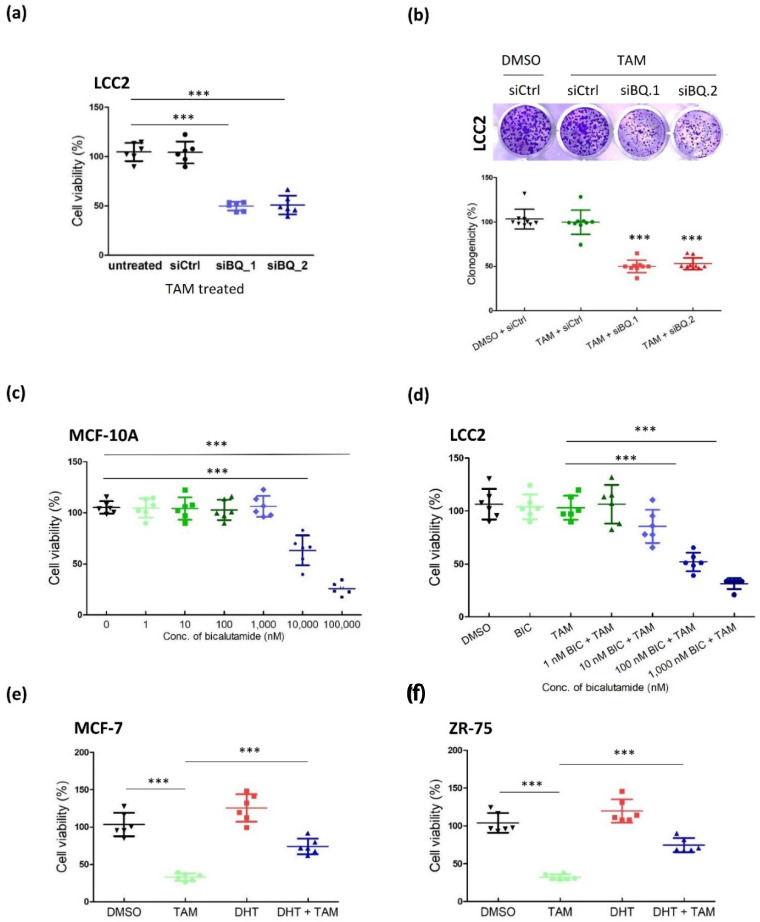
Inhibition of AR could reverse tamoxifen resistance. (**a**) Knockdown of BQ could recover tamoxifen sensitivity as revealed by MTT assay. LCC2 was transfected with 25 µM of non-targeting siRNA (siCtrl), siBQ.1 or siBQ.2. 48 h post-transfection, the cells were treated with 4 µM of tamoxifen (4-OHT; TAM) for 96 h. MTT assay was employed to determine cell viability. Results were shown as mean ± SD from 6 independent experiments. (**b**) Knockdown of BQ could recover tamoxifen sensitivity as revealed by clonogenic assay. 48 h post-transfection, the cells were treated with 4 µM of tamoxifen (4-OHT; TAM) for 14 days. 0.01% crystal violet was used to stain the cells. Results were shown as mean ± SD from 9 independent experiments. (**c**) The effect of AR antagonist bicalutamide on cell viability of MCF-10A. The cells were treated with different concentrations of bicalutamide for 96 h. MTT assay was employed to determine cell viability. Results were shown as mean ± SD from 6 independent experiments. (**d**) Dosage dependent effect of bicalutamide on reversing tamoxifen resistance in LCC2. The cells were treated with4 µM of tamoxifen (4-OHT; TAM) and different concentrations of bicalutamide (BIC) for 96 h. MTT assay was employed to determine cell viability. Results were shown as mean ± SD from 6 independent experiments. Activation of AR could decrease the efficacy of tamoxifen in (**e**) MCF-7 and (**f**) ZR-75. The cells were treated with 4 µM of tamoxifen (4-OHT; TAM) 0.1 nM of dihydrotestosterone (DHT; androgen) for 96 h. MTT assay was employed to determine cell viability. Results were shown as mean ± SD from 6 independent experiments. One-way ANOVA was employed. Bonferroni’s multiple comparison test was employed to determine the significance between 2 groups. *** represent *p* < 0.001.

Next, we assessed whether AR activity could modulate tamoxifen resistance by comparing the response to tamoxifen in control LCC2 and LCC2 with BQ down-regulation. First, we confirmed that knockdown of BQ within 96 h did not affect cell viability (Figure S2). Next, we performed an MTT assay to determine if knockdown of BQ would affect tamoxifen response. The results from MTT suggested that knockdown of BQ could make LCC sensitive to tamoxifen (Figure 2a). A Clonogenic assay revealed similar results (Figure 2b). To further consolidate our findings, we employed AR antagonist bicalutamide (BIC). We found that 1 µM of BIC was the maximal non-lethal dosage of bicalutamide in a normal breast cell line MCF-10A as revealed by the MTT assay (Figure 2c). Next, we confirmed that neither 1 µM of bicalutamide nor 4 µM of tamoxifen affected the cell viability of LCC2 (Figure 2d). However, co-treatment of different concentrations of bicalutamide and 4 µM of tamoxifen could recover response to tamoxifen in LCC2 in a dose-dependent manner (Figure 2d). As further confirmation, we treated MCF-7 and ZR-75 with 0.1 nM of DHT to activate AR signaling and determine the response to tamoxifen in these activated cells. The results from MTT showed that activation of AR made MCF-7 (Figure 2e) and ZR-75 (Figure 2f) tolerant to tamoxifen, suggesting that activation of AR signaling can confer tamoxifen resistance in breast cancer.

### 3.2. Identification of IL-8 as a Candidate to Modulate Tamoxifen Resistance in Breast Cancer

**Figure 3:**
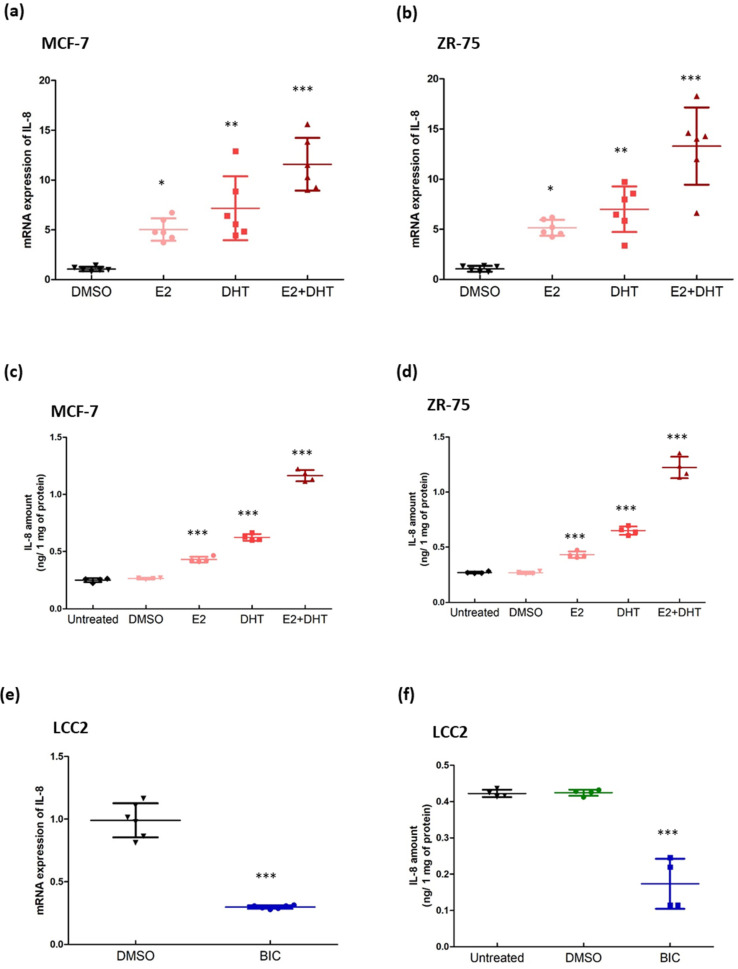
Modulating the activity of AR could interfere with the expression of IL-8. The effect of estrogen and androgen on the expression of IL-8 in (**a**) MCF-7 and (**b**) ZR-75. The cells were treated with 1 nM of Estradiol (E2; estrogen) and/or 0.1 nM of dihydrotestosterone (DHT; androgen) for 24 h. qPCR was performed. Actin was used as the internal control. Results were shown as mean ± SD from 6 independent experiments. One-way ANOVA was employed. Bonferroni’s multiple comparison test was employed to determine the significance between DMSO and any of the treatment groups. (**c**,**d**) ELISA was performed to confirm the effect of 1 nM of E2 and 0.1 nM of DHT on the production of IL-8. Culture medium was collected after 24 h of the treatment. ELISA was performed to determine the amount of IL-8 in the medium. Results were shown as mean ± SD from 4 independent experiments. One-way ANOVA was employed. Bonferroni’s multiple comparison test was employed to determine the significance between untreated and any of the treatment groups. Suppression of AR could reduce the (**e**) mRNA expression and (**f**) protein production of IL-8 in LCC2. The cells were treated with 1 µM of bicalutamide (BIC; AR antagonist) for 48 h. qPCR was performed to determine mRNA. Results were shown as mean ± SD from 4 independent experiments. Student’s *t*-test was employed to determine statistical significance. ELISA was performed to evaluate the production of IL-8 in the culture medium. Results were shown as mean ± SD from 4 independent experiments. One-way ANOVA was employed. Bonferroni’s multiple comparison test was employed to determine the significance between untreated and BIC treated groups. *, **, and *** represent *p* < 0.05, *p* < 0.01 and *p* < 0.001.

NCOR2 is a repressor for both ER and AR. We hypothesized that BQ overexpression, by compromising the repressor activity of NCOR2, would enhance the activities of both ERE and ARE in breast cancer, conferring resistance to tamoxifen. Through in silico analysis, we found 22 genes that contained both ARE and ERE in their promoter regions (Figure S3). As confirmation, we treated MCF-7 with either 1 nM of E2 or 1 nM of DHT. We used qPCR to determine the expression of these genes. The results showed that only interleukin-8 (IL-8) was responsive to both E2 and DHT (Figure S3). Co-treatment of 1 nM of E2 and 1 nM of DHT further enhanced IL-8 expression in MCF-7 (Figure 3a) and ZR-75 (Figure 3b). Results from ELISA also confirmed enhanced production of IL-8 protein by this treatment (Figure 3c,d). When we treated LCC2 cells with bicalutamide, the expression of IL-8 was reduced, as revealed by qPCR (Figure 3e) and ELISA (Figure 3f), thus confirming that IL-8 expression in breast cancer can be governed by ARE and ERE activity.

**Figure 4:**
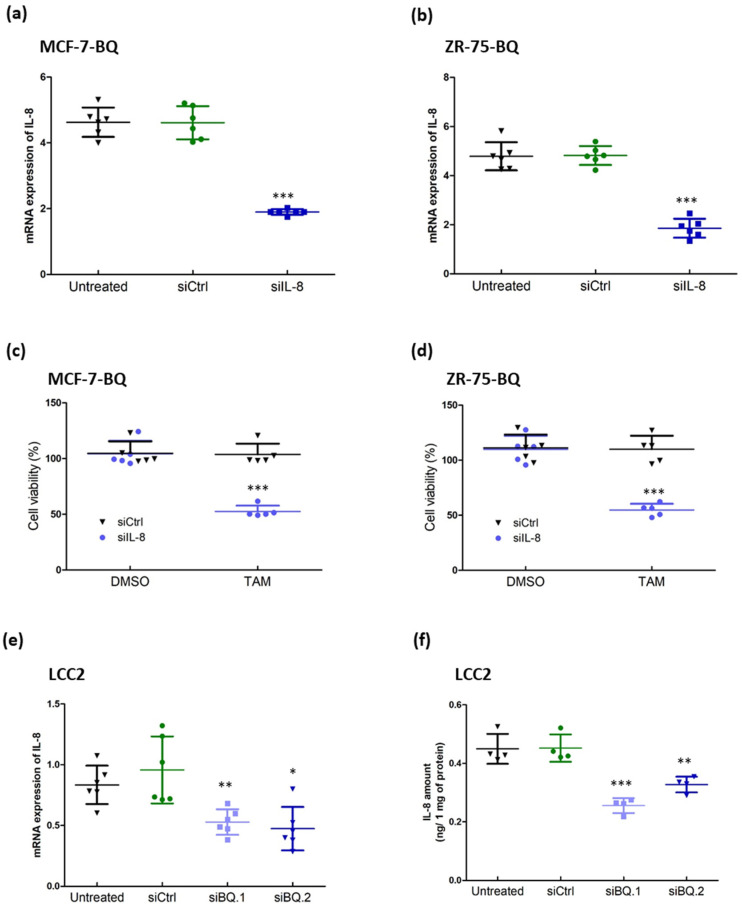
Modulating the expression of IL-8 could alter the response to tamoxifen. Knockdown efficiency of siRNA against IL-8 in (**a**) MCF-7-BQ and (**b**) ZR-75-BQ. The cells were treated with 25 µM of non-targeting siRNA (siCtrl) or IL-8 specific siRNA (siIL-8). qPCR was performed 48 h post-transfection. Actin was used as the internal control. Results were shown as mean ± SD from 6 independent experiments. One-way ANOVA was employed. Bonferroni’s multiple comparison test was employed to determine the significance between untreated and siIL-8 treated groups. Knockdown of IL-8 could reverse tamoxifen resistance in (**c**) MCF-7-BQ and (**d**) ZR-75-BQ. The cells were transfected with siCtrl and siIL-8. 4 µM of 4-OHT (TAM) was used after 48 h of the transfection. MTT assay was performed to determine cell viability after 72 h of TAM treatment. Results were shown as mean ± SD from 5 independent experiments. Student’s *t*-test was employed to determine statistical significance between siCtrl and siIL-8 treated groups. Knockdown of BQ could reduce the (**e**) mRNA expression and (**f**) protein expression of IL-8 in LCC2. LCC2 cells were treated with the siRNAs. qPCR was performed to determine the mRNA level of IL-8, 48 h post-transfection. Results were shown as mean ± SD from 6 independent experiments. ELISA was performed to determine the amount of IL-8 in the culture medium. Results were shown as mean ± SD from 4 independent experiments. One-way ANOVA was employed. Bonferroni’s multiple comparison test was employed to determine the significance between untreated and siRNAs treated groups. *, **, and *** represent *p* < 0.05, *p* < 0.01 and *p* < 0.001.

We next examined whether IL-8 could modulate tamoxifen response. First, we confirmed that the expression of IL-8 mRNA (Figure S4a) and the amount of IL-8 protein (Figure S4b) were higher in LCC2 when compared to MCF-7. Next, we found that overexpression of BQ in MCF-7 and ZR-75 could enhance the expression of IL-8 on both mRNA (Figure S4c,d) and protein levels (Figure S4e,f). The expression of IL-8 was directly correlated with the expression of BQ, suggesting that BQ regulates the expression of IL-8. We employed siRNA to reduce the expression of IL-8 in tamoxifen-resistant cell lines, MCF-7-BQ (Figure 4a) and ZR-75-BQ (Figure 4b). We found that down-regulation of IL-8 could reverse tamoxifen resistance in these cell lines (Figure 4c,d). When we knocked down BQ expression in LCC2, IL-8 expression was significantly reduced, as revealed by qPCR (Figure 4e) and ELISA (Figure 4f). As previously shown, BQ knockdown made LCC2 sensitive to tamoxifen (Figure 2A). These results suggest that IL-8 is one of the downstream pathways of BQ overexpression that modulates resistance to tamoxifen in breast cancer.

### 3.3. IL-8 Activated the AKT-ERK1/2 Axis to Modulate the Response to Tamoxifen

**Figure 5:**
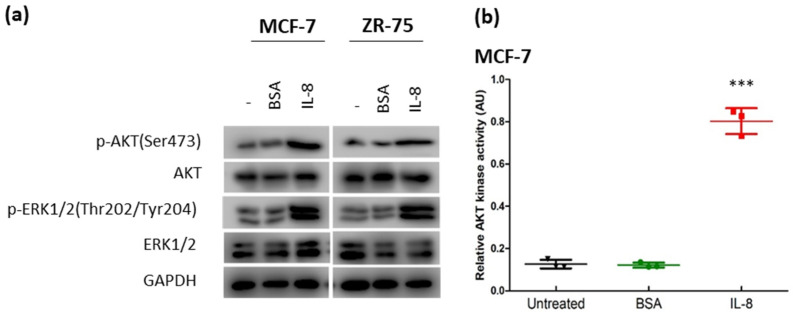

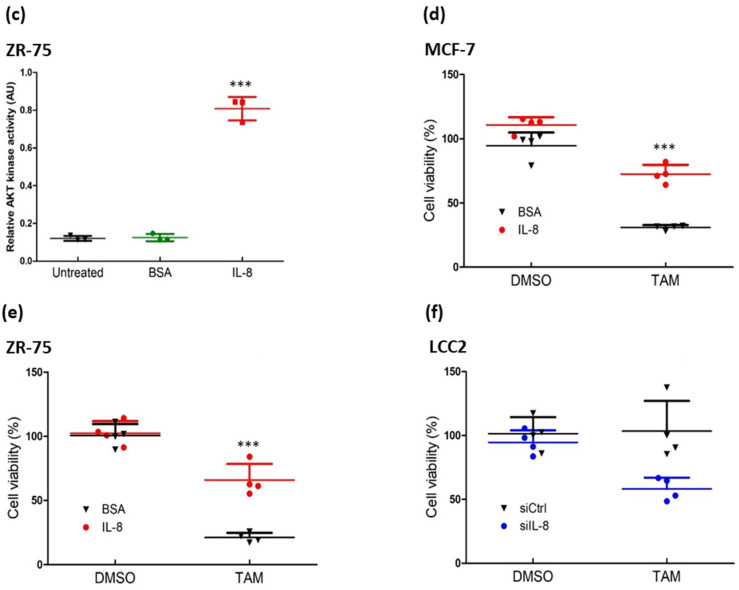
Treatment of IL-8 could induce tamoxifen resistance. (**a**) IL-8 could activate AKT and ERK1/2 in MCF-7 and ZR-75 cells. The cells were treated with 5 ng/mL of IL-8. Proteins were harvested 24 h post-treatment. Western blot was employed to determine the expression of the candidate proteins. GAPDH was the loading control. The treatment of IL-8 could enhance AKT kinase activity in (**b**) MCF-7 and (**c**) ZR-75. The cells were treated with 5 ng/mL of IL-8. AKT kinase activity assay was performed after 24 h of the treatment. Results were shown as mean ± SD from 3 independent experiments. One-way ANOVA was employed. Bonferroni’s multiple comparison test was employed to determine the significance between untreated and IL-8 treated groups. IL-8 treatment could enhance the tolerance to tamoxifen in (**d**) MCF-7 and (**e**) ZR-75. The cells were co-treated with 4 µM of 4-OHT (TAM) and 5 ng/mL of IL-8 or BSA for 96 h. MTT assay was employed to determine cell viability. Results were shown as mean ± SD from 4 independent experiments. Student’s *t*-test was employed to determine statistical significance between BSA, and IL-8 treated groups. (**f**) Knockdown of IL-8 could reduce tamoxifen resistance in LCC2. The cells were transfected with 25 µM of siCtrl or siIL-8. The cells were treated with 4 µM of TAM after 48 h of the transfection. MTT assay was performed after 96 h of TAM treatment. Results were shown as mean ± SD from 4 independent experiments. Student’s *t*-test was employed to determine statistical significance between siCtrl and siIL-8 treated groups. *** represents *p* < 0.001.

IL-8 (CXCL8) is a pro-inflammatory chemokine that plays an essential role in inflammation and tumor progression [25,26]. IL-8 exerts its effects by binding to the specific G protein-coupled receptors of CXCR1 and CXCR2 [27]. The binding of IL-8 would activate a series of kinases, leading to the activation of the AKT-ERK1/2 signaling cascade [28,29]. Activation of AKT-mediated signaling cascades is associated with the development of tamoxifen resistance [9]. CXCR1 interacts specifically with IL-8, while CXCR2 can bind with different cytokines [30]. We confirmed that IL-8 treatment could activate the AKT-ERK1/2 axis, as revealed by Western blot in MCF-7 and ZR-75 (Figure 5a; Figure S6b). Moreover, IL-8 treatment could enhance AKT kinase activity (Figure 5b,c) and confer tamoxifen resistance (Figure 5d,e). In contrast, tamoxifen resistance was reversed by IL-8 knockdown in LCC2 (Figure 5f). These results demonstrated that IL-8 would be essential for tamoxifen resistance in breast cancer.

### 3.4. Targeting CXCR1/2 Could Reverse Tamoxifen Resistance in Breast Cancer In Vitro and In Vivo

**Figure 6:**
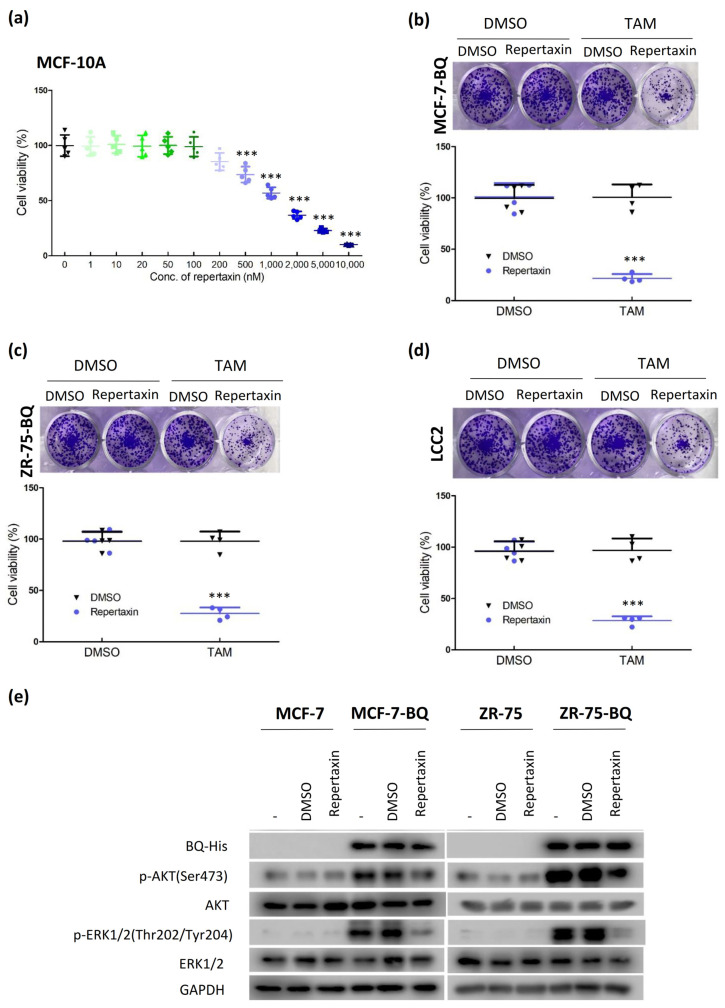
Treatment of CXCR1/2 inhibitor, repertaxin could reduce tamoxifen resistance. (**a**) The dosage-dependent effect of repertaxin on cell viability of MCF-10A. Non-cancerous breast epithelial cell line MCF-10A was used. The cells were treated with different concentrations of repertaxin for 96 h. MTT assay was performed. Results were shown as mean ± SD from 5 independent experiments. One-way ANOVA was employed. Bonferroni’s multiple comparison test was employed to determine the significance between 0 nM and other concentrations. Repertaxin could reduce tamoxifen resistance in (**b**) MCF-7-BQ, (**c**) ZR-75-BQ and (**d**) LCC2. The cells were co-treated with 4 µM of 4-OHT and 0.1 µM of repertaxin for 14 days. Clonogenic assay was performed. 0.01% crystal violet was used to stain the colonies. Results were shown as mean ± SD from 4 independent experiments. Student’s *t*-test was employed to determine statistical significance between DMSO and repertaxin treated groups. *** represents *p* < 0.001. (**e**) Repertaxin could suppress AKT and ERK1/2 activation on BQ overexpressing cells. MCF-7, MCF-7-BQ, ZR-75 and ZR-75-BQ cells were treated with 0.1 µM of repertaxin for 48 h. Western blot was used to determine the expression of the protein candidates. GAPDH was used as the loading control.

Repertaxin is a non-competitive allosteric inhibitor of CXCR1/2. To examine whether treatment of repertaxin would reverse tamoxifen resistance, we first confirmed that 100 nM of repertaxin was the maximal non-lethal dosage of repertaxin in MCF-10A (Figure 6a) and employed this concentration to test whether this drug would reverse tamoxifen resistance. Clonogenic assay from MCF-7-BQ (Figure 6b), ZR-75-BQ (Figure 6c), and LCC2 (Figure 6d) confirmed that repertaxin could reverse tamoxifen resistance. Moreover, Western blot demonstrated that repertaxin reduced the levels of p-AKT and p-ERK1/2 in BQ overexpressing cell lines (Figure 6e; Figure S6c).

**Figure 7:**
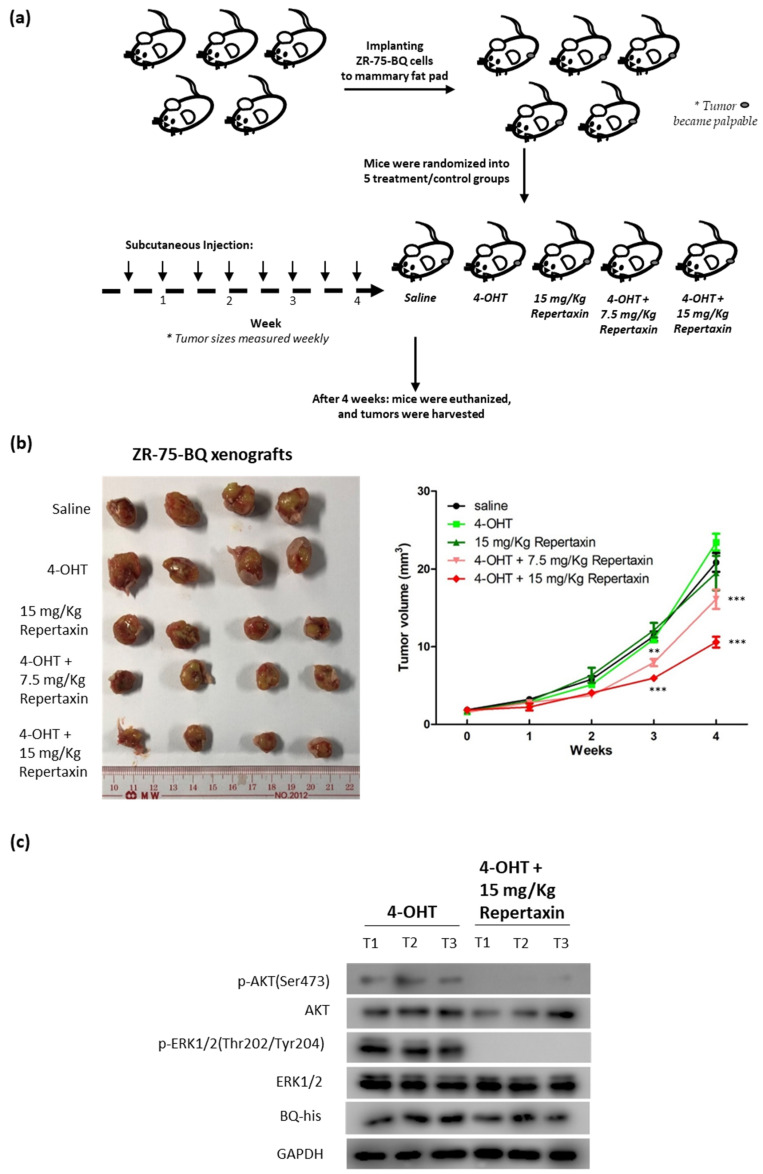
Repertaxin could reverse tamoxifen resistance in vivo. (**a**) ZR-75-BQ cell line was employed to establish xenografts. The cells were implanted onto the mammary fat pad of nude mice. The mice received saline, tamoxifen (4-OHT; 500 mg; twice per week), repertaxin (15 mg/Kg; twice per week), tamoxifen + repertaxin (500 mg of 4-OHT + 7.5 mg/Kg of repertaxin; twice per week) and tamoxifen + repertaxin (500 mg of 4-OHT + 15 mg/Kg of repertaxin; twice per week). Repertaxin was delivered by subcutaneous injection. After 4 weeks of treatment, tumors were harvested. (**b**) The photo showed the effect of different treatments on tumor size. Results were shown as mean ± SD from 4 independent tumors. Two-way ANOVA was performed. Bonferroni’s multiple comparison test was employed to determine the significance between saline and other treatment groups at each time point. ** and *** represent *p* < 0.01 and *p* < 0.001, respectively. (**c**) Treatment of repertaxin could reduce the levels of activated AKT and ERK1/2 in the tumors. Proteins were harvested from the tumors. Western blot was performed to analyze the expression of the indicated protein candidates in 3 of the independent tumors. GAPDH was used as the loading control.

To further confirm the effect, an in vivo model was employed. Xenografts were established using ZR-75-BQ cells implanted onto the mammary fat pad of female nude mice. The mice were treated with tamoxifen (0.5 mg/mouse; twice/week) and given subcutaneous injection of repertaxin (7.5 mg/Kg and 15 mg/Kg; twice/week; Figure 7a). Compared with the saline control, mice treated with either 4-OHT or repertaxin alone did not show significant tumor reduction (Figure 7b). Mice treated with repertaxin and 4-OHT together could significantly suppress tumor growth, with such an effect being dose-dependent as revealed by two-way ANOVA (Figure 7b; F = 8.58; *p* < 0.001). Western blot analysis confirmed that treatment of repertaxin could reduce the levels of both p-AKT and p-ERK1/2 in the tumors (Figure 7c; Figure S6d). Our results, therefore, suggest that targeting CXCR1 by repertaxin could recover tamoxifen sensitivity.

### 3.5. Clinical Significance of CXCR1 in Breast Cancer

**Figure 8:**
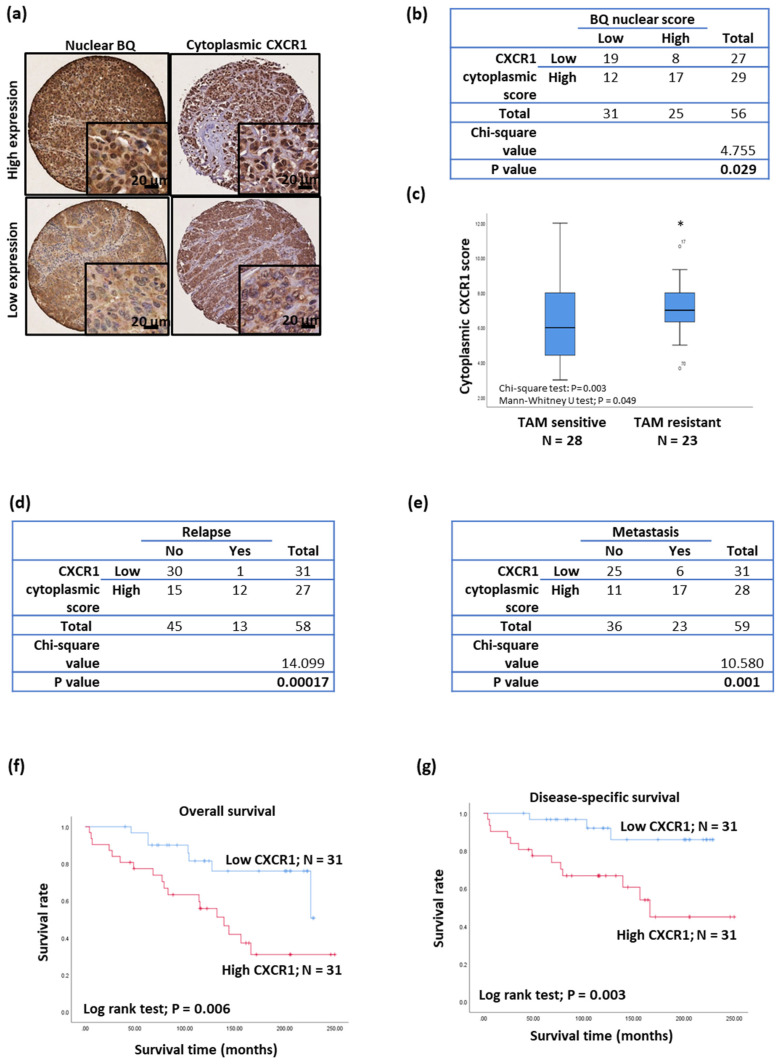
Clinical significance of CXCR1 in breast cancer. (**a**) Immunohistochemistry of BQ and CXCR2 was performed on primary ER+ve breast tumor on TMA. (**b**) Chi-square test to determine the correlation between nuclear BQ and cytoplasmic CXCR1. (**c**) Tamoxifen resistance was associated with high expression of cytoplasmic CXCR1. Chi-square test and Mann–Whitney U test were employed. * represents *p* < 0.05. Chi-square test to determine the correlation of cytoplasmic CXCR1 with (**d**) relapse and (**e**) metastasis. High expression of CXCR1 was associated with poorer (**f**) overall survival and (**g**) disease-specific survival in ER+ve breast cancer. Log-rank test was employed.

Immunohistochemistry was employed to assess the expression of CXCR1 and BQ in the primary ER+ve breast tumor (Figure 8a). Cytoplasmic CXCR1 expression was positively correlated with nuclear BQ expression (Figure 8b) shown by the chi-square test, *p* = 0.029. High cytoplasmic CXCR1 expression was also associated with tamoxifen resistance, relapse, and metastasis (Figure 8c–e). The Kaplan–Meir log-rank text showed it was significantly associated with poor outcome for overall survival (log-rank test; *p* = 0.006; Figure 8f) and disease-specific survival (log-rank test; *p* = 0.003; Figure 8g). Univariate Cox regression analysis for overall survival (Table 1) showed that cases with high cytoplasmic CXCR1 were significantly associated with poorer overall survival (RR = 3.171, 95% CI 1.322, 7.610; *p* = 0.010), but this failed to remain significant on multivariate analysis. Similar findings were obtained for combined analysis of high cytoplasmic CXCR1 and high nuclear BQ expression. Interestingly, a Cox regression analysis for disease-specific survival (Table 2) showed cases with high cytoplasmic CXCR1 were significantly associated with poorer disease-specific survival both for univariate (RR = 5.350, 95% CI 1.519, 18.840; *p* = 0.009) and multivariate analyses (RR = 4.661, 95% CI 1.313, 16.545; *p* = 0.017). Similar findings were also obtained for a combined analysis of high cytoplasmic CXCR1 and high nuclear BQ, with significant association with poorer disease-specific survival in both univariate (RR = 5.401, 95% CI 1.500, 19.449; *p* = 0.010) and multivariate analyses (RR = 4.860, 95% CI 1.318, 17.919; *p* = 0.018). These results confirm that cytoplasmic CXCR1 expression could be an independent prognostic factor for disease-specific survival in ER+ve breast cancer.

## 4. Discussion

Most breast cancer patients are ER+ve and receive tamoxifen as adjuvant endocrine treatment. Despite the high efficacy of tamoxifen, one-third of these patients still relapse after tamoxifen treatment. In this study, we found that BQ, in disrupting the gene repressor function of NCOR2, could induce the expression of IL-8. We confirmed the presence of functional ERE and ARE in the IL-8 promotor. Excess BQ could compete with the repressor function of NCOR2, reducing its suppressive effect on ER and AR. Activation of both ERE and ARE in the IL-8 promotor intensifies BQ’s effect on IL-8 production. Targeting the IL-8 mediated signaling cascade could reverse tamoxifen resistance in vitro and in vivo, illustrating one more possible way to combat tamoxifen resistance in breast cancer.

Our demonstration that BQ overexpression can promote ARE and ERE activities in ER+ve breast cancer cell lines is novel. BQ overexpression can thus enhance both AR and ER-mediated signaling activities that can lead to tamoxifen resistance. Through in silico analysis, we identified 22 candidate genes that contained both ARE and ERE within the promoter region and confirmed that interleukin-8 (IL-8) had both functional ARE and ERE activity (Figure S1), suggesting that IL-8 could be regulated by both AR and ER in ER+ve breast cancer. Having shown that BQ overexpression can enhance both ERE activity [14] and ARE activity (Figure 1a–e), we next confirmed that stimulating ERE and ARE activity can enhance the expression of IL-8 in ER+ve breast cancer cell lines (Figure 3a–d). Inhibition of AR reduced IL-8 expression in the BQ overexpressing cell lines (Figure 3e–f). These results suggest that the modulation of IL-8 expression by BQ involved AR. We confirmed that IL-8 could activate both AKT and ERK1/2 via CXCR1 to confer tamoxifen resistance; however, we cannot exclude the effect of CXCR2 as repertaxin targets both CXCR1 and CXCR2. The small inhibitor repertaxin could reverse tamoxifen resistance in vitro and in vivo. Our study illustrates one more possible way to combat tamoxifen resistance in breast cancer.

IL-8 is a pro-inflammatory chemokine that has been suggested to promote tumor progression, angiogenesis, and metastasis in cancer [31]. Overexpression of IL-8 is associated with drug resistance. Inhibition of IL-8 has been suggested to reverse paclitaxel and doxorubicin resistance in breast cancer cell lines [32]. It has been reported that the expression of IL-8 was significantly increased in tamoxifen-resistant MCF-7 and ZR-75 cell lines [33]. IL-8 interacts with its receptor CXCR1 to activate downstream signaling pathways [34], such as the AKT [35] and ERK1/2 [36]. Activation of PI3K/AKT has been suggested to induce tamoxifen resistance in ER+ve breast cancer [37], and increased activity of the ERK1/2 pathway has been shown to involve tamoxifen resistance [38]. Furthermore, IL-8 has been demonstrated to activate STAT3 signaling in prostate cancer for promoting the disease progression [39]. Our previous study demonstrated that overexpression of BQ could enhance STAT3 signaling by up-regulating the expression of IL-6 to modulate tamoxifen resistance in breast cancer [22]. Overexpression of BQ might mediate IL-6 and IL-8 signaling; in addition, their crosstalk might contribute to the drug resistance. Our study is the first to describe AR mediating tamoxifen resistance through enhancing the expression of IL-8, which subsequently activates the AKT and ERK1/2 signaling cascade. Furthermore, we showed that repertaxin could compromise AKT and ERK1/2 activities in vitro (Figure 6) and in vivo (Figure 7). Apart from CXCR1, CXCR2 has been shown to modulate the development, progression and drug response of breast cancer [40]. CXCR2 can also modulate drug resistance. Activation of CXCR2 by IL6 might induce resistance to paclitaxel, while activation of CXCR2 by IL8 might induce resistance to doxorubicin [32]. In our study, we found that repertaxin could reduce tamoxifen resistance. Hence, CXCR1/2 can be a possible target for developing a therapeutic agent against tamoxifen resistance in breast cancer.

For ER+ve breast cancer, ER signaling plays a critical role in disease progression. Activation of ER by its ligand estrogen can trigger transcription of target genes that maintain estrogen response element (ERE), leading to cancer cell growth and proliferation. Therefore, targeting ER is an efficient approach to inhibit ER+ breast cancer. In this study, we revealed that AR could also contribute to tamoxifen resistance. There are 70–90% of breast cancer patients who express the AR [41], with several studies indicating that AR might be a predictive or prognostic factor and a drug target in breast cancer [42]. However, it is still controversial whether AR is a good or bad prognostic factor. AR has been shown to correlate with favorable outcomes, such as smaller tumor size, lower tumor grade, less necrosis, lower Ki-67 levels, and better treatment response in ER+ve breast cancer [43,44,45]. However, the AR-to-ER expression ratio has been considered to affect the prognosis and response to tamoxifen treatment. If the ratio of AR to ER was greater than 2, it had an increased risk of failure with tamoxifen therapy [46]. These findings suggest the role of AR could be context-dependent and should not be regarded as an independent factor contributing to tamoxifen resistance per se. Our study suggests that AR might contribute to intensifying signaling components that modulate tamoxifen resistance. Although the role of AR remains controversial, it is nevertheless an attractive candidate for developing novel breast cancer therapies, potentially improving breast cancer patients’ survival outcomes.

## 5. Conclusions

In summary, we demonstrated a possible mechanism mediated by BQ to induce tamoxifen resistance in ER+ve breast cancer through AR-mediated signaling. BQ could up-regulate IL-8 to modulate tamoxifen resistance and confer tamoxifen resistance through IL-8 mediated AKT and ERK1/2 pathways. Targeting IL-8 and its receptors would be a potential therapeutic approach to combat tamoxifen resistance in ER+ve breast cancer.

## Figures and Tables

**Table 1:** Cox regression analysis of overall survival on ER+ breast cancer. Values bolded represents statistical significance.

Clinical-Pathological Parameters	Univariate Analysis	
	**RR (95% CI)**	***p* value**
Age (*n* = 69)	1.682 (0.785, 3.602)	0.181
T-stage (*n* = 30)	5.522 (1.226, 24.871)	**0.026**
Lymph-node involvement (*n* = 63)	0.981 (0.438, 2.197)	0.962
Tumor-Grade (*n* = 68)	1.389 (0.637, 3.027)	0.409
Histological type (*n* = 69)	1.368 (0.323, 5.795)	0.671
HER2 status (*n* = 49)	1.11 (0.427, 2.888)	0.83
Tumor size (*n* = 52)	0.938 (0.377, 2.334)	0.89
Cases with Hi-CXCR1 cytoplasm score (*n* = 62)	3.171 (1.322, 7.61)	**0.01**
Cases with both Hi-CXCR1 & BQ score (*n* = 36)	3.205 (1.107, 9.276)	**0.032**
**Clinical-pathological parameters**	**Multivariate analysis**	
	**RR (95% CI)**	***p* value**
T-stage (*n* = 26)	8.332 (1.363, 50.943)	**0.022**
Cases with Hi-CXCR1 cytoplasm score (*n* = 26)	3.265 (0.805, 13.247)	0.098
**Clinical-pathological parameters**	**Multivariate analysis**	
	**RR (95% CI)**	***p* value**
T-stage (*n* = 17)	9.31 (1.016, 85.311)	**0.048**
Cases with both Hi-CXCR1 & BQ score (*n* = 17)	5.053 (0.796, 32.066)	0.086

**Table 2:** Cox regression analysis of disease-specific survival on ER+ breast cancer. Values bolded represents statistical significance.

Clinical-Pathological Parameters	Univariate Analysis	
	**RR (95% CI)**	***p* value**
Age (*n* = 69)	1.37 (0.543, 3.456)	0.505
T-stage (*n* = 30)	3.695 (0.67, 20.372)	0.133
Lymph-node involvement (*n* = 63)	1.373 (0.497, 3.796)	0.541
Tumor-Grade (*n* = 68)	3.463 (1.139, 10.525)	**0.029**
Histological type (*n* = 69)	0.81 (0.186, 3.527)	0.778
HER2 status (*n* = 49)	1.715 (0.499, 5.893)	0.392
Tumor size (n = 52)	1.341 (0.403, 4.46)	0.632
Cases with Hi-CXCR1 cytoplasm score (*n* = 62)	5.35 (1.519, 18.84)	**0.009**
Cases with both Hi-CXCR1 & BQ score (*n* = 36)	5.401 (1.5, 19.449)	**0.01**
**Clinical-pathological parameters**	**Multivariate analysis**	
	**RR (95% CI)**	***p* value**
Tumor-Grade (*n* = 61)	3.113 (0.879, 11.026)	0.078
Cases with Hi-CXCR1 cytoplasm score (*n* = 61)	4.661 (1.313, 16.545)	**0.017**
**Clinical-pathological parameters**	**Multivariate analysis**	
	**RR (95% CI)**	***p* value**
Tumor-Grade (*n* = 36)	1.642 (0.447, 6.04)	0.455
Cases with both Hi-CXCR1 & BQ score (*n* = 36)	4.86 (1.318, 17.919)	**0.018**

## Data Availability

Transcriptional Regulatory Element Database (http://rulai.cshl.edu/cgi-bin/TRED/tred.cgi?process=home; assessed on 18 May 2019) was used to identify genes with androgen response element (ARE) and estrogen response element (ERE). The datasets and materials generated in this study are available from the corresponding author on reasonable request.

## Supplementary Materials

The following supporting information can be downloaded at https://www.mdpi.com/article/10.3390/life12010093/s1, Figure S1. Endogenous BQ expression level and basal ARE activity in MCF-7 and LCC2, Figure S2. The effect of BQ knockdown in LCC2, Figure S3. Relative mRNA expression of genes contained both ARE and ERE in their promoter region, Figure S4. Expression of IL-8 in MCF-7 and LCC2 and the effect of BQ overexpression in MCF-7 and ZR-75, Figure S5. Protein quantification of Western blot in this study, Figure S6. Original blots of Western blot of this study. Table S1: List of qPCR primer sequences used in this study, Table S2: Clinical characteristics of breast cancer patients.
