# Correction: Tsoi et al. Overexpression of BQ323636.1 Modulated AR/IL-8/CXCR1 Axis to Confer Tamoxifen Resistance in ER-Positive Breast Cancer. *Life* 2022, *12*, 93

**DOI:** 10.3390/life14081044

**Published:** 2024-08-22

**Authors:** Ho Tsoi, Ling Shi, Man-Hong Leung, Ellen P. S. Man, Zi-Qing So, Wing-Lok Chan, Ui-Soon Khoo

**Affiliations:** 1 Department of Pathology, Li Ka Shing Faculty of Medicine, The University of Hong Kong, Hong Kong, China; tsoiho@hku.hk (H.T.); u3005146@connect.hku.hk (L.S.); george09@connect.hku.hk (M.-H.L.); ellenman@hku.hk (E.P.S.M.); u3569644@connect.hku.hk (Z.-Q.S.); 2 Department of Clinical Oncology, Li Ka Shing Faculty of Medicine, The University of Hong Kong, Hong Kong, China; winglok@hku.hk

## Error in Figure

In the original publication (doi: 10.3390/life12010093; [1]), there was a mistake in Figure 8a as published. We provided photomicrograph captions to illustrate what high- and low- nuclear BQ expressing breast cancer samples in tissue microarray look like (Figure 8a). For the high nuclear BQ expression, we inadvertently made use of the same case we had previously chosen as illustration in our previous publication (Figure 4A, PMID: 29420220), as this was probably the best illustrative core for high nuclear BQ expression. The purpose of these figures is merely illustrative, providing an example of high-and low-staining samples, without deriving from it any biological data or information. In order not to infringe on potential copyright issues, relating to our previous publication PMID: 29420220, we now replace high BQ expression with a new figure. The corrected Figure 8a appears below.

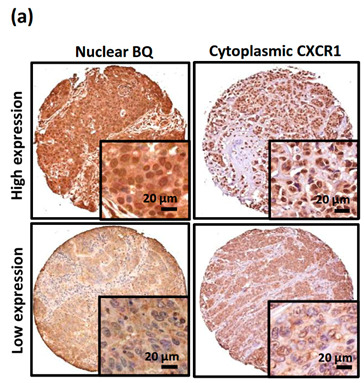



The authors state that the scientific conclusions are unaffected. This correction was approved by the Academic Editor. The original publication has also been updated.
