# Supplementary material for: Overexpression of BQ323636.1 Modulated AR/IL-8/CXCR1 Axis to Confer Tamoxifen Resistance in ER-Positive Breast Cancer
Source: Life (Basel). 2022 Jan 10;12(1):93. doi: 10.3390/life12010093 (PMC8778777; doi:10.3390/life12010093)
Supplement: Supplementary file 1 [file life-12-00093-s001.zip › life-1526063-supplementary.pdf]

life

MDPI

Supplementary

Overexpression of BQ323636.1 Modulated AR/IL-8/CXCR1 Axis to Confer Tamoxifen Resistance in ER-Positive Breast Cancer

Ho Tsoi [1], Ling Shi [1], Man-Hong Leung [1], Ellen P.S. Man [1], Zi-Qing So [1], Wing-Lok Chan [2] and Ui-Soon Khoo [1,*]

1 Department of Pathology, Li Ka Shing Faculty of Medicine, The University of Hong Kong, Hong Kong, China; tsoiho@hku.hk (H.T.); u3005146@connect.hku.hk (L.S.); george09@connect.hku.hk (M.-H.L.); ellenman@hku.hk (E.P.S.M.); u3569644@connect.hku.hk (Z.-Q.S.)

2 Department of Clinical Oncology, Li Ka Shing Faculty of Medicine, The University of Hong Kong, Hong Kong, China; winglok@hku.hk" winglok@hku.hk (W.-L.C.)

* Correspondence: uskhoo@pathology.hku.hk" uskhoo@pathology.hku.hk (U.-S.K.)

Table S1. List of qPCR primer sequences used in this study.

Gene	Forward primer (5'-->3')	Reverse primer (5'-->3')
SP1	ATCATCCGGACACCAACAGT	TGATTGTTTGGGCTTGTGGG
PSG1	AGAGCAAGACCCACTCTGTT	TTGGTGAGTTCTGAGTGGCT
ITGA2	GCTCATCCAAAGTTGCCACA	TAGAGCAAGATGGGGTGTGG
NOS3	TGGCTTTCCCTTCCAGTTCC	TGGACAGATGTGAGAAGGCA
UBE2L3	TCCTCCAGTCCTTCTCCTCA	AGGCCAGAGGTCAGTTACAC
FBLN1	TTGCTCCTGACCGTCAAGAT	AGGAAAGCAACAGGAGGGAG
CYP27B1	TGTAGGCACAAGACCAAGGT	TTCTCACCTGGCTTCCTGAG
CDKN1A	GGGCTGGGAGTAGTTGTCTT	AGCCGAGAGAAAACAGTCCA
TGFA	CGTGAGCCCTCGGTAAGTAT	AGCAACAAACCAACCAAGCA
NT5E	ACCCCTCCAATTCCTTCCTC	GCAGGAAGAGTGGAGAGGTT
ITGB1	GAGTGCCGTAACAACTGTGG	TGCCCTAAAGCTACCTAACTGT
MYB	GCATGGATCCTGTGTTTGCA	TCAAAAGTTCAGTGCTGGCC
SRC	CCTCCTTCCCCGTACTTTGT	AGGCACTCTTTTCCCTCCTC
CGA	ACCATACCACTTTGACACGC	AGGCTTTATTTGCAGTGGAACA
EGFR	AACAGTCCTGCTCCTCAACC	TTACGCCCTTCACTGTGTCT
IL8	TAGCCAGGATCCACAAGTCC	TGCTTCCACATGTCCTCAC
HSPB1	CTCAAACGGGTCATTGCCAT	GACTCTGCTCTGGACGTCTG
NOL3	CTGAATCGGATGCCACCAAG	AACAGCCAGAATCGTGGAGA
APOE	TCAAGAGCTGGTTCGAGCC	TTCGGCGTTCAGTGATTGTC
SERPINE1	AAGCCTAATCAGCCCACCAT	TAGCATTTGACACCACCCCT
NR3C2	AGCACGTTTTCTGGCTTCTG	CCTCCTCTCCCACATGTGTT

Table S2. Clinical characteristics of breast cancer patients.

Clinical characters		Number of Cases	Percentage (%)
**Breast cancer patients**		**137**	**100**
**Age**	**<56**	**65**	**47.4**
	**>=56**	**72**	**52.6**
**T stage**	**I, II**	**41**	**29.9**
	**III, IV**	**10**	**7.3**
**Lymph Node status**	**Positive**	**67**	**48.9**
	**Negative**	**56**	**40.9**
**Tumor Grade**	**1, 2**	**49**	**35.8**
	**3**	**74**	**54.0**
**Tumor Size**	**<2 cm**	**37**	**27.0**
	**>=2 cm**	**56**	**40.9**
**Estrogen Receptor status**	**Positive**	**71**	**51.8**
	**Negative**	**25**	**18.2**
**Progesterone receptor status**	**Positive**	**49**	**35.8**
	**Negative**	**37**	**27.0**
**HER2 receptor status**	**Positive**	**35**	**25.5**
	**Negative**	**37**	**27.0**
**Triple Negative status**	**Positive**	**13**	**9.5**
	**Negative**	**71**	**51.8**

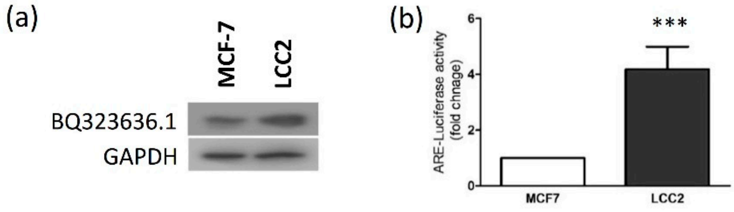

Figure S1. Endogenous BQ expression level and basal ARE activity in MCF-7 and LCC2. (a) Comparison of endogenous BQ expression level in MCF-7 and LCC2. Western blot was employed to determine the expression of BQ. GAPDH was used as the loading control. (b) Comparison of basal ARE activity in MCF-7 and LCC2. Luciferase reporter assay with androgen response element (ARE) was employed. Results were shown as mean ± SD from 6 independent experiments. Student's t-test was employed to determine statistical significance. *** represents $p < 0.001$.

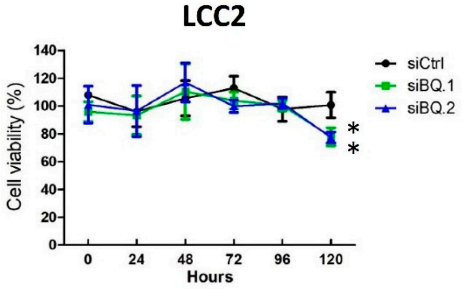

Figure S2. The effect of BQ knockdown in LCC2. LCC2 cells were treated with 25 µM of non-targeting siRNA (siCtrl), siBQ.1 or siBQ.2 for the indicated periods. MTT assay was employed to determine cell viability. Untreated LCC2 at each of the time points was used as the reference. Results were shown as mean ± SD from 3 independent experiments. Two-way ANOVA with Bonferroni post-test was employed to determine statistical significance with the treatment group and siCtrl. * represents $p < 0.05$. .

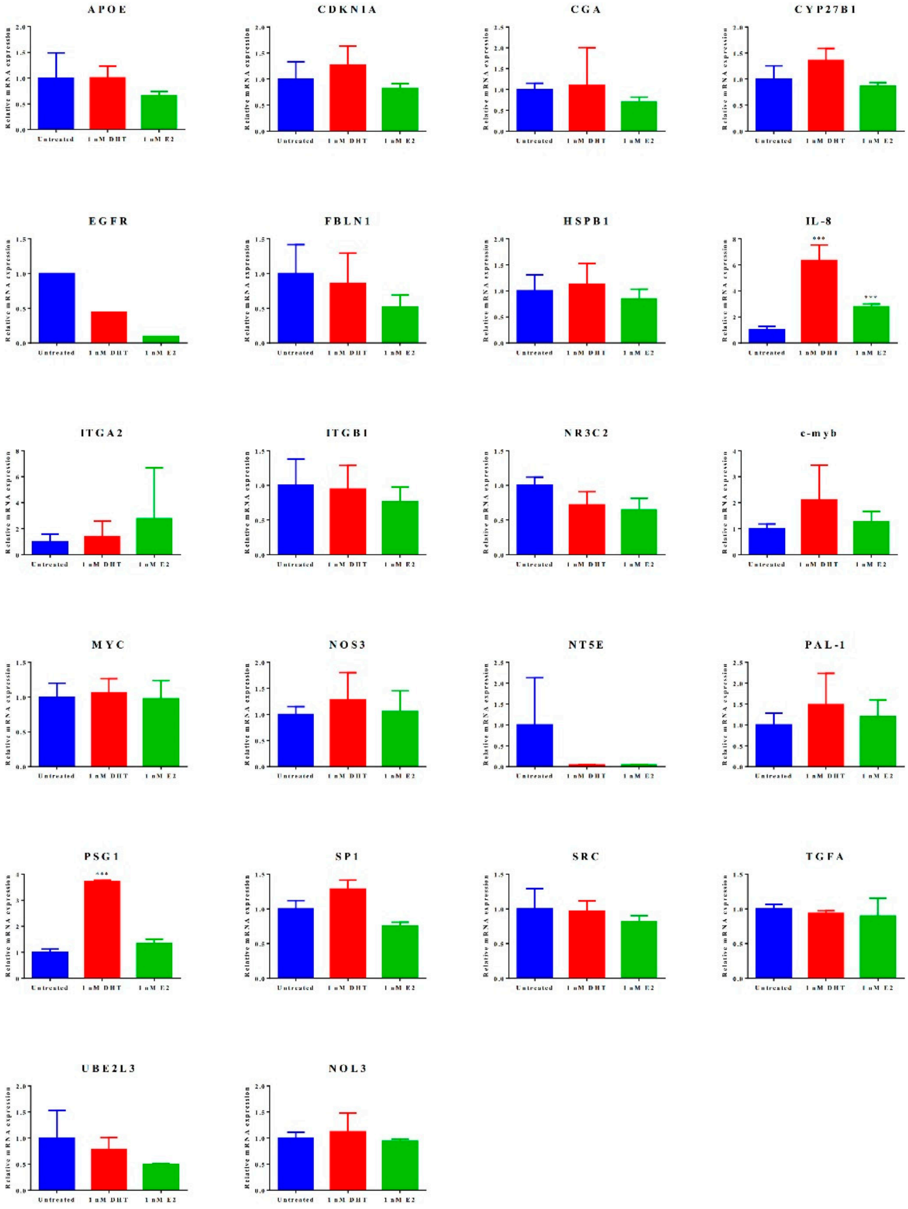

Figure S3. Relative mRNA expression of genes contained both ARE and ERE in their promoter region. (a) MCF-7 cells were treated with 1 nM dihydrotestosterone (DHT, Red) , 1 nM estradiol (E2, Green) or untreated (Blue) for 24 h. (b) ZR-75 cells were treated with 1 nM DHT, 1 nM E2 or untreated for 24 h. The relative mRNA expression of each gene was shown as mean±SD. Statistical significance was determined by student's t-test. *** $p < 0.001$.

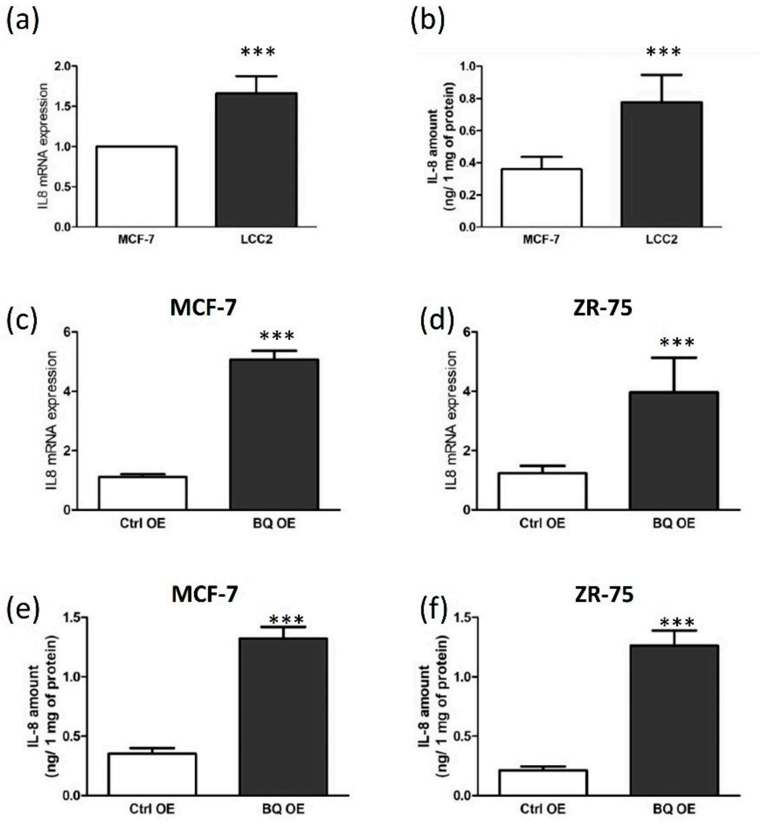

Figure S4. Expression of IL-8 in MCF-7 and LCC2 and the effect of BQ overexpression in MCF-7 and ZR-75. Comparing the expression of IL-8 in LCC2 and MCF-7 on (a) mRNA and (b) protein levels. Comparing the effect of BQ overexpression on IL-8 on mRNA levels in (c) MCF-7 and (d) ZR-75. Comparing the effect of BQ overexpression on IL-8 on protein levels in (e) MCF-7 and (f) ZR-75. qPCR was employed to determine the expression of IL-8. Actin was used as the internal control. ELISA was employed to determine IL-8 protein in the cell lysates. Results were shown as mean ± SD from 6 independent experiments. Student's t-test was employed to determine statistical significance. *** represents $p < 0.001$.

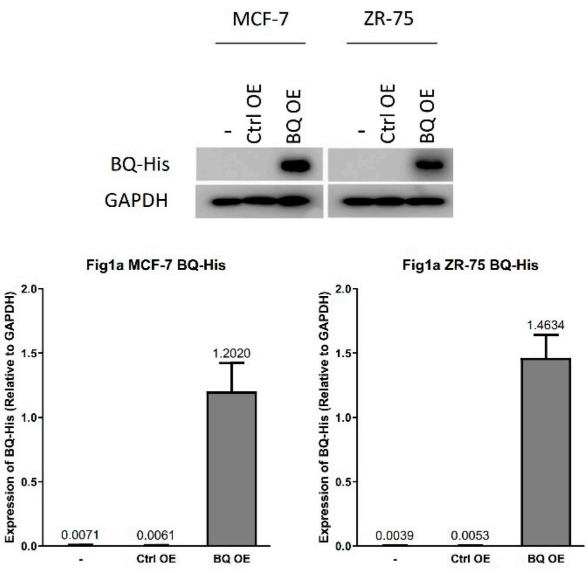

Figure S5. a. Protein Quantification is performed by Image J from Western blot in Fig 1a.

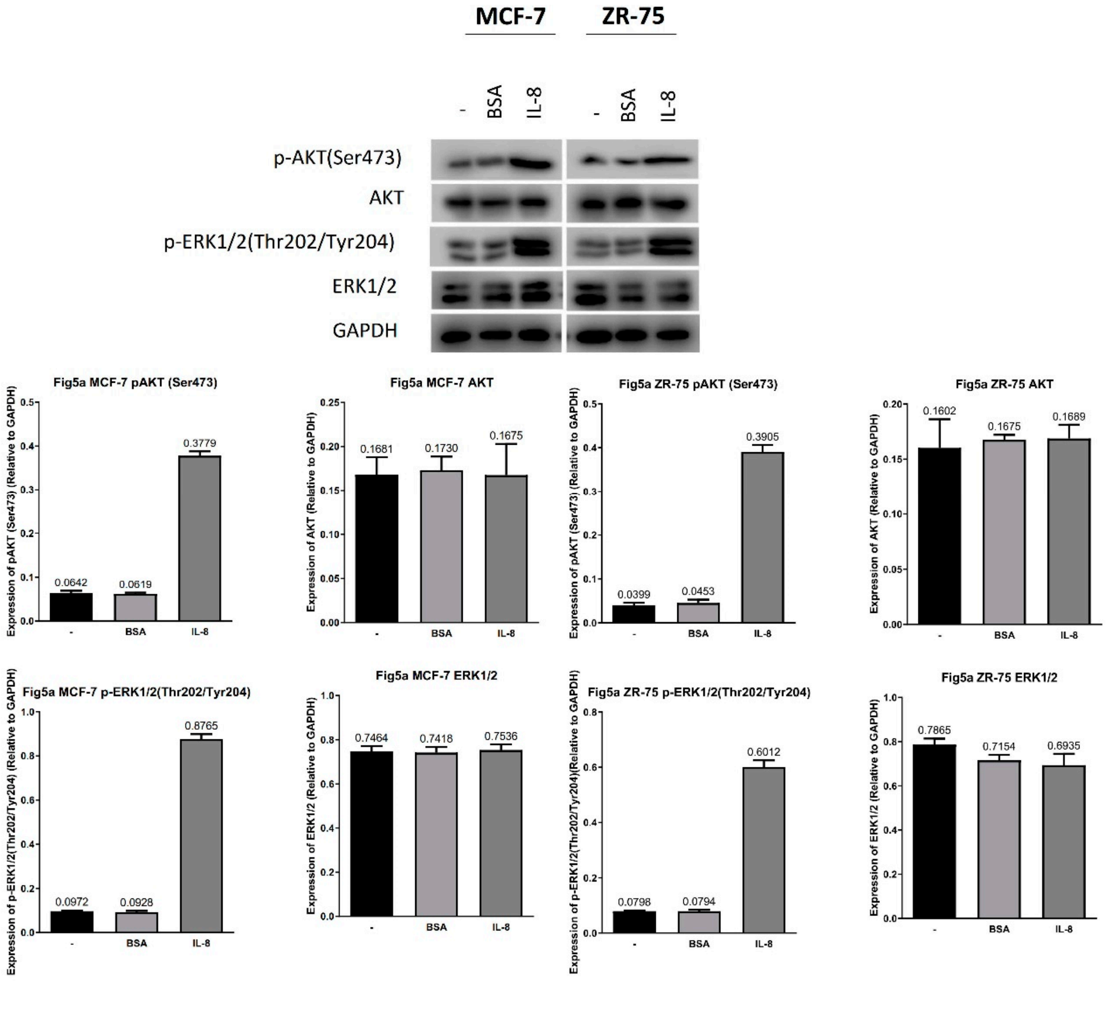

Figure S5. b. Protein Quantification is performed by Image J from Western blot in Fig 5a.

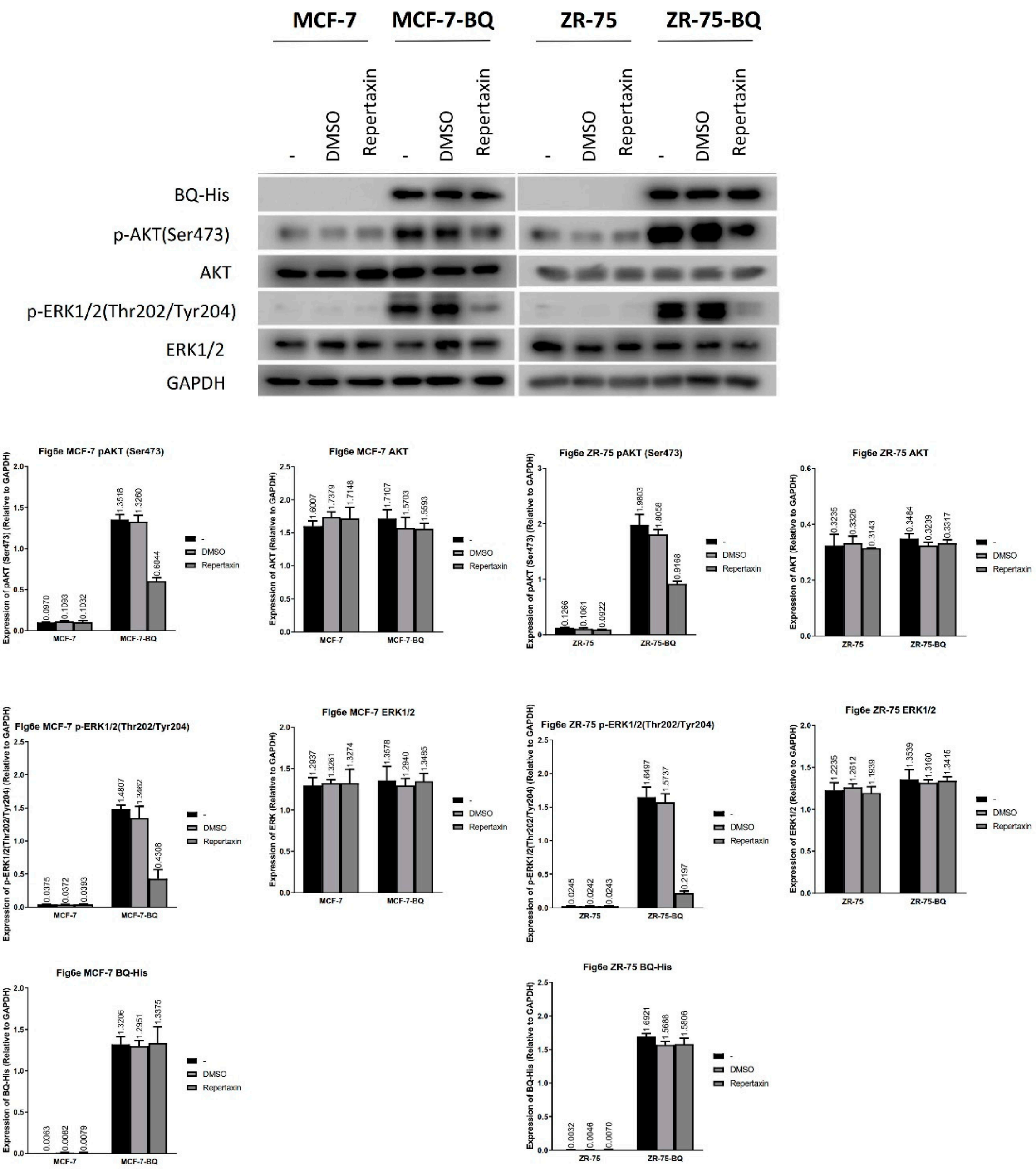

Figure S5. c. Protein Quantification is performed by Image J from Western blot in Fig 6e.

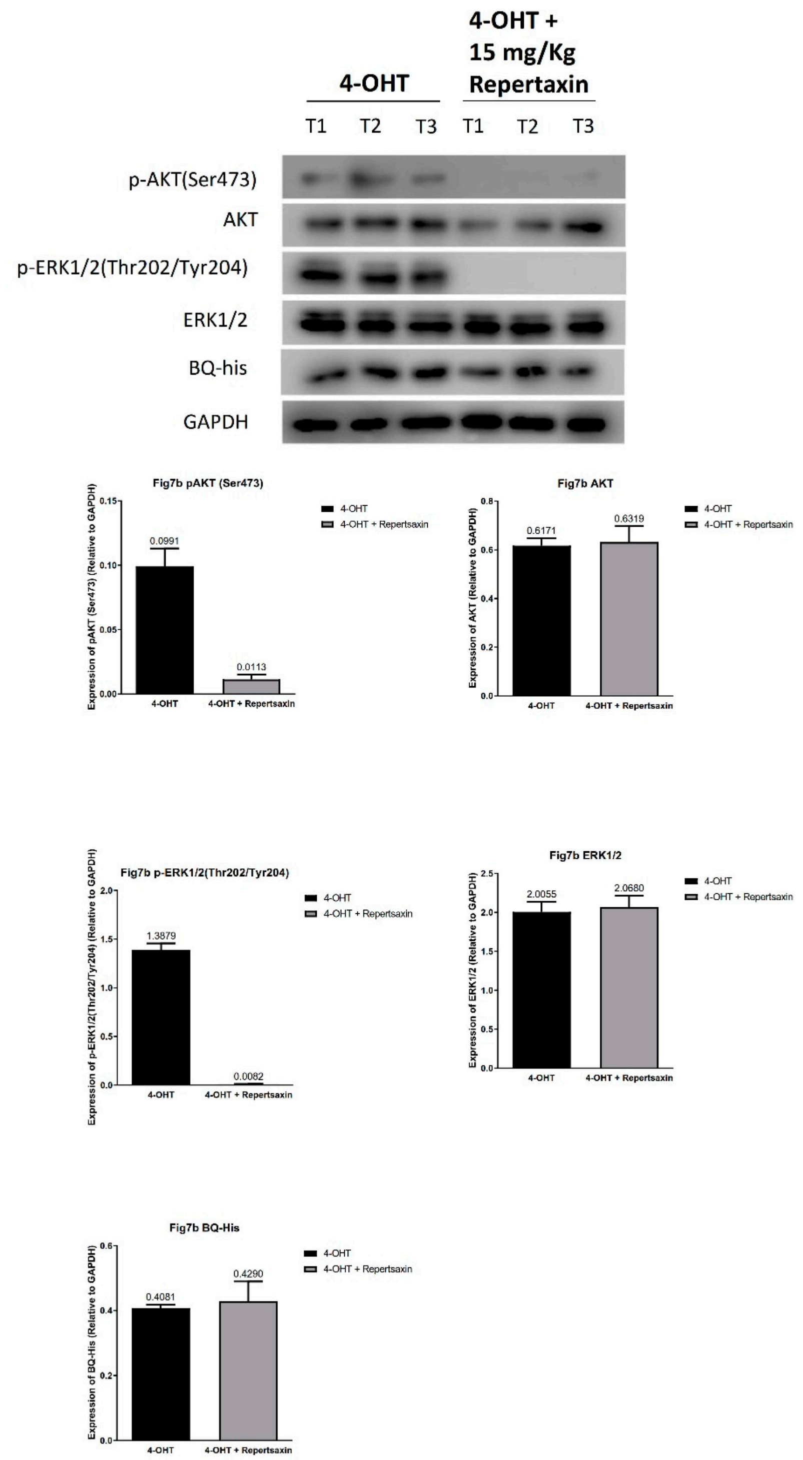

Figure S5. d. Protein Quantification is performed by Image J from Western blot in Fig 7b.

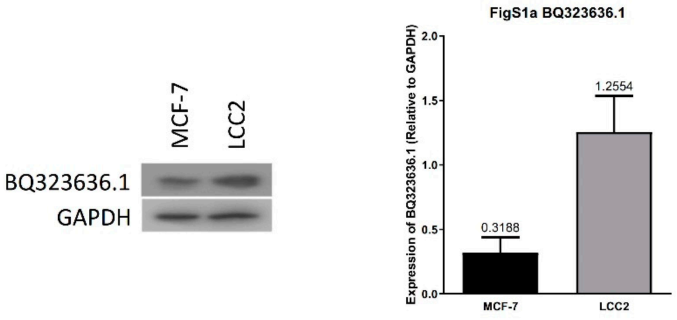

Figure S5. e. Protein Quantification is performed by Image J from Western blot in Fig S1a.

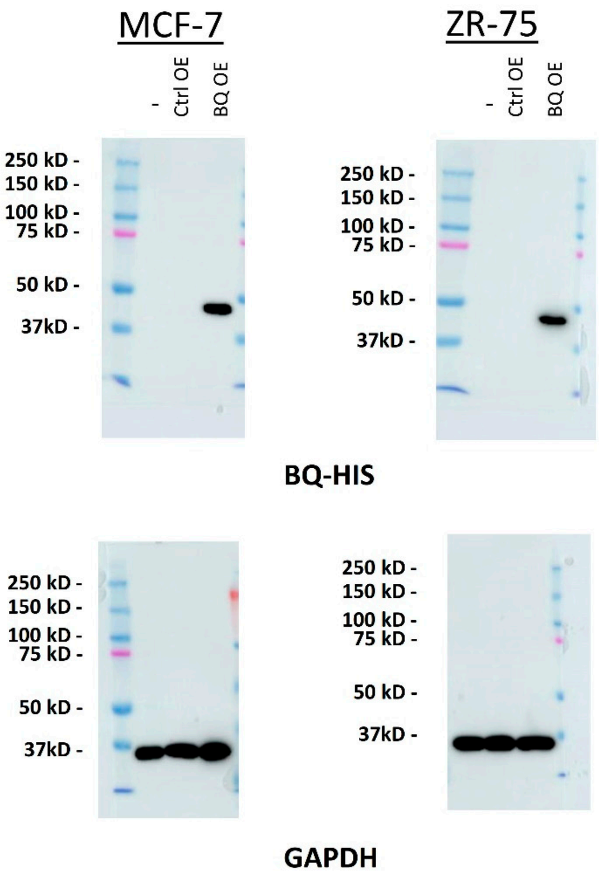

Figure S6. a. Original blots for the cropped/composite for Fig 1a.

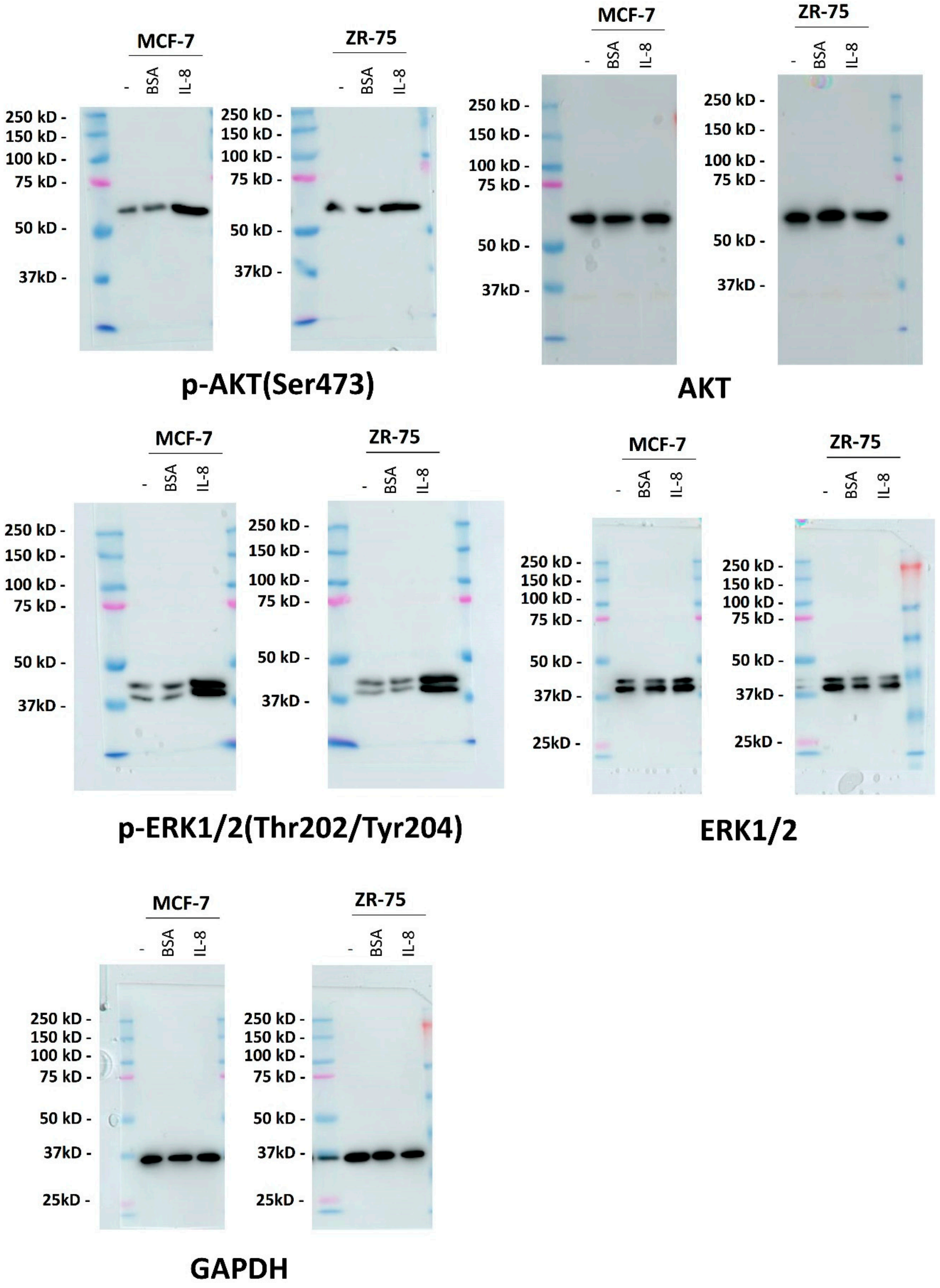

Figure S6. b. Original blots for the cropped/composite for Fig 5a.

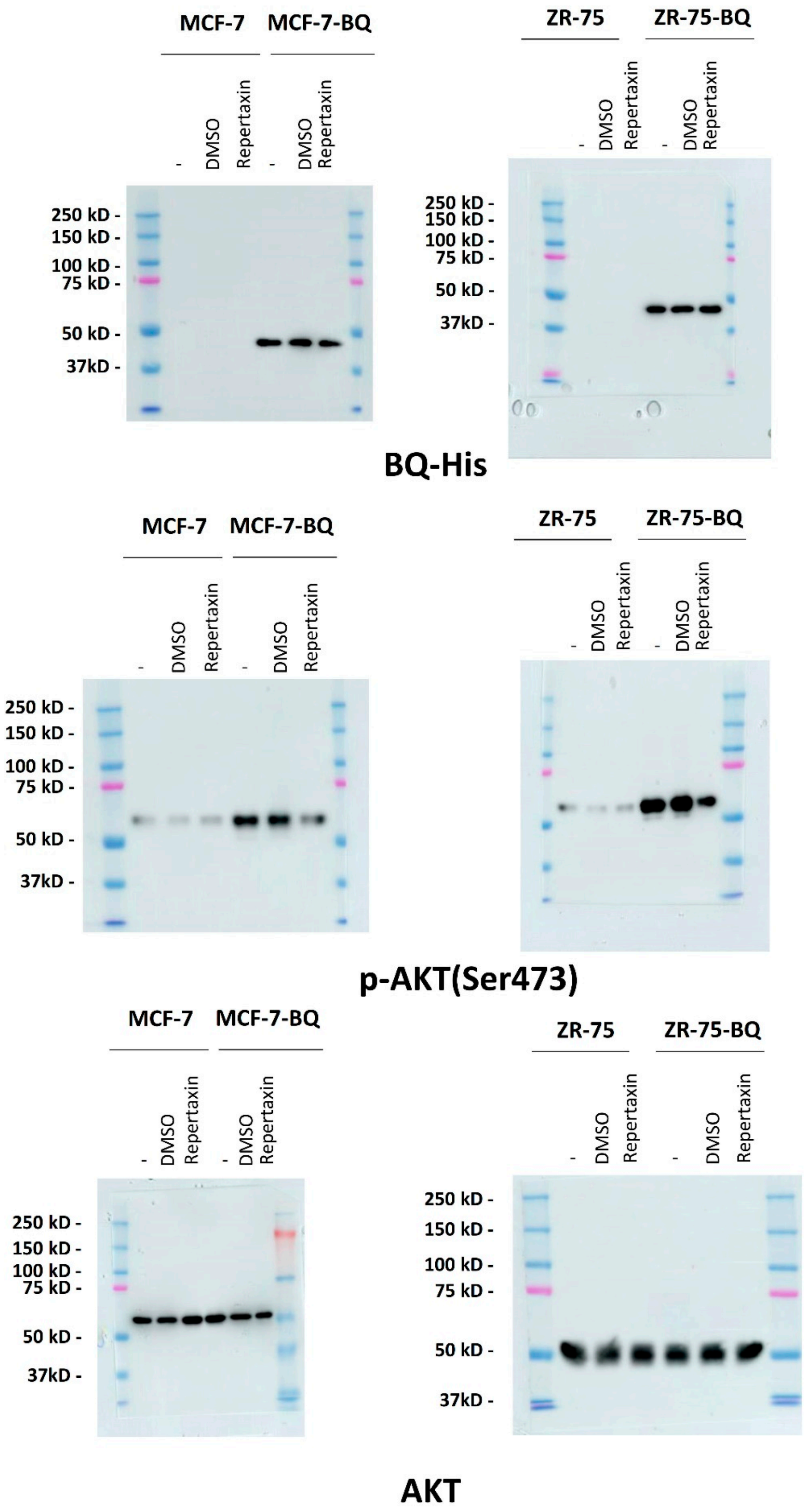

Figure S6. c. Original blots for the cropped/composite for Fig 6e.

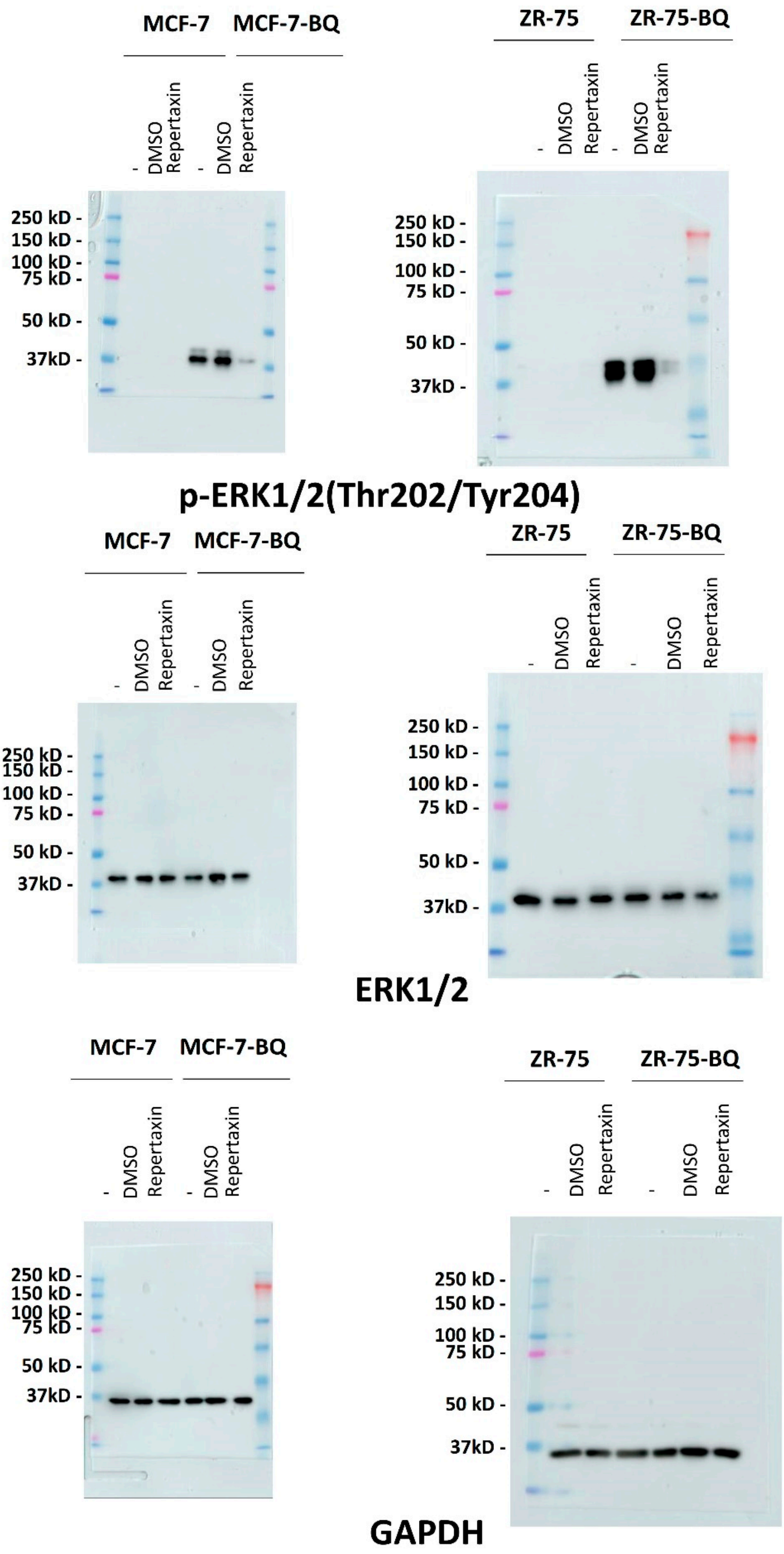

Figure S6. c. Original blots for the cropped/composite for Fig 6e.

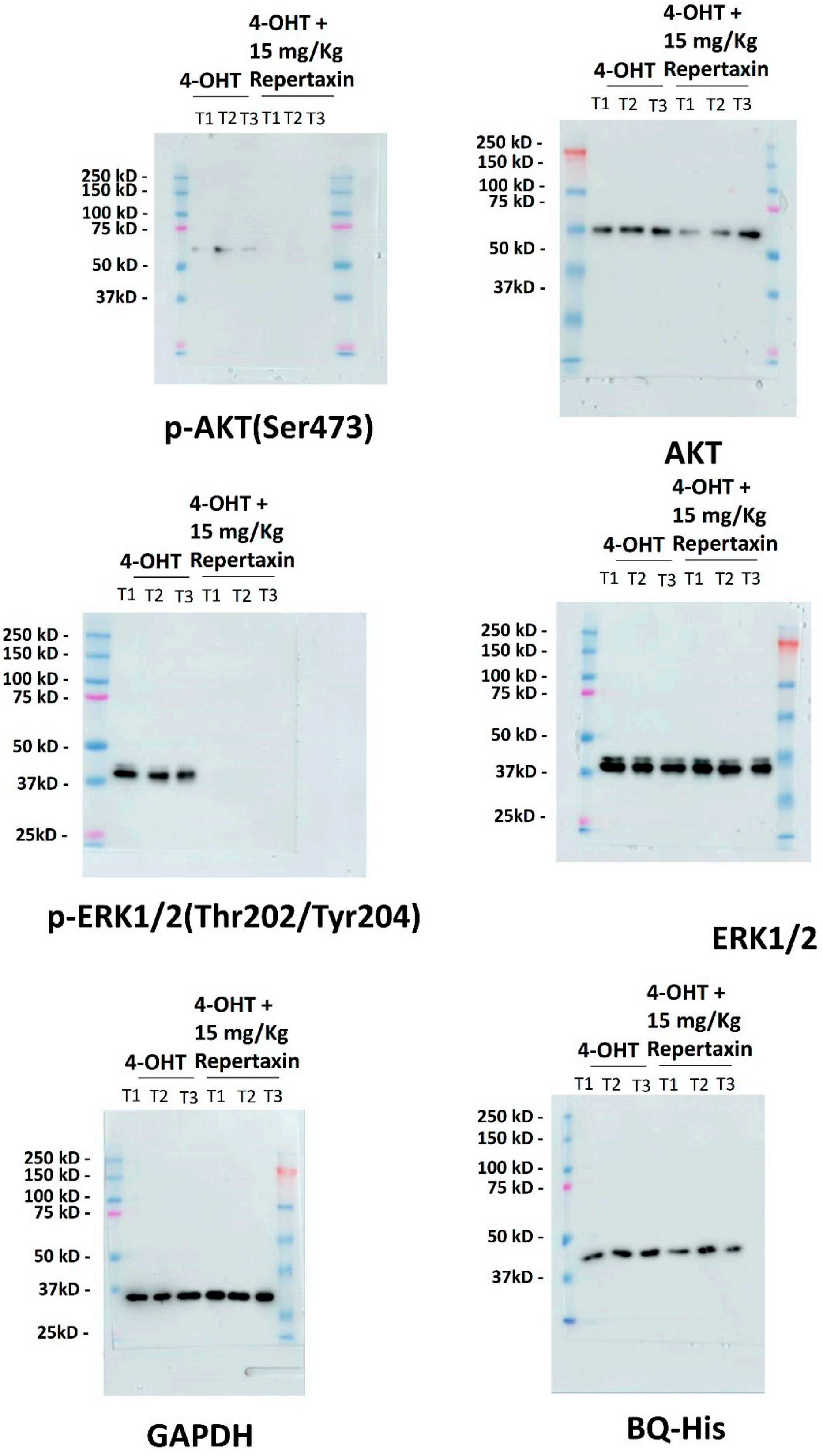

Figure S6. d. Original blots for the cropped/composite for Fig 7b.

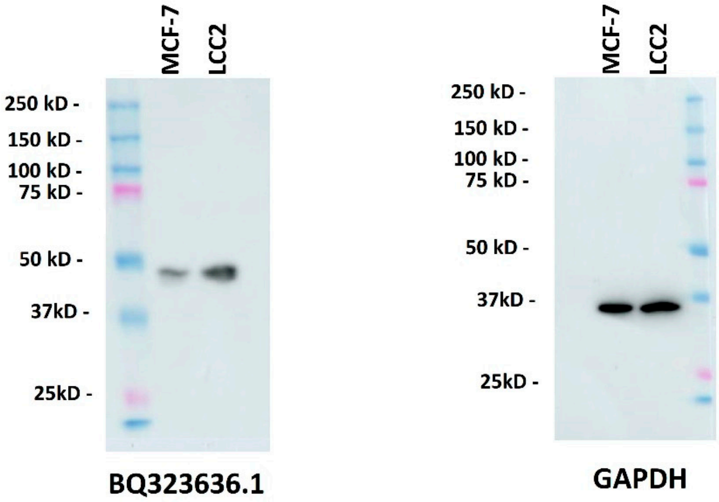

Figure S6. e. Original blots for the cropped/composite for Fig S1a.
